# Efficacy and safety of Xingnaojing injection for post-operative patients of intracerebral haemorrhage: a meta-analysis and systematic review

**DOI:** 10.3389/fphar.2024.1411026

**Published:** 2024-06-05

**Authors:** Yanbo Song, Fangbiao Xu, Shuliang Li, Yongkang Sun, Xinzhi Wang

**Affiliations:** ^1^ Center of Encephalopathy, The First Affiliated Hospital of Henan University of Chinese Medicine, Zhengzhou, Henan, China; ^2^ First Clinical Medical College, Henan University of Chinese Medicine, Zhengzhou, Henan, China

**Keywords:** Xingnaojing injection, traditional Chinese medicine, Chinese patent medicine, injection, intracerebral haemorrhage, post-operative patients, meta-analysis

## Abstract

**Background:**

Intracerebral haemorrhage (ICH) is the deadliest subtype of stroke. Surgery remains a vital measure for life-saving in emergency situations, however, the recovery of post-operative patients is not optimistic. This study aimed to evaluate the evidence of the efficacy and safety of Xingnaojing injection (XNJ) for post-operative patients of ICH.

**Methods:**

From inception to 31 January 2024, we searched eight representative databases for randomized controlled trials on post-operative patients of ICH treated with XNJ. A meta-analysis was conducted using R4.2.2, and the quality of the evidence was evaluated by GRADE criteria.

**Results:**

The results indicated that the combination of XNJ with conventional western medicine therapy improved the total efficiency rate (RR = 1.26; 95% CI [1.21 to 1.32]; *p* < 0.0001), reduced the all-cause mortality within 15 days (RR = 0.45; 95% CI [0.30 to 0.67]; *p* < 0.0001), decreased the volume of hematoma (MD = −4.72; 95% CI [-7.43 to −2.01]; *p* = 0.0006) and perihematomal edema (MD = −4.11; 95% CI [-8.11 to −0.11]; *p* = 0.0441), reduced the TNF-α levels (SMD = −1.61, 95% CI [−2.23 to −0.99], *p* < 0.0001), decreased neurological impairment (SMD = −1.44; 95% CI [-1.78 to −1.11]; *p* < 0.0001), improved the activities of daily living (SMD = 1.22; 95% CI [0.78 to 1.66]; *p* < 0.0001), and enhanced the consciousness level (MD = 2.08, 95% CI [1.22 to 2.93], *p* < 0.0001). In addition, the complications of the combination therapy group were lower (RR = 0.43; 95% CI [0.35 to 0.54]; *p* < 0.0001) and the adverse drug reactions were comparable to the control group (RR = 0.89; 95% CI [0.55 to 1.45]; *p* = 0.6521). The trial sequential analysis results showed that the sample size is sufficient.

**Conclusion:**

Current evidence indicates that XNJ can enhance the efficiency, reduce mortality, and lower the incidence of complications, while demonstrating good tolerability of post-operative patients of ICH. However, the level of evidence from existing studies is relatively weak, and only prove short-term effects, and high-quality RCTs are needed to further verify the accuracy of these conclusions.

**Systematic Review Registration:** identifier (PROSPERO 2024 CRD42024503006). https://www.crd.york.ac.uk/prospero/display_record.php?ID=CRD42024503006, Identifier CRD42024503006.

## 1 Introduction

Intracerebral haemorrhage (ICH) refers to non-traumatic spontaneous intracerebral haemorrhage (NBCMA and CDGNBCMA, 2019), which falls under the category of hemorrhagic stroke in Traditional Chinese Medicine (TCM) (Wang and Gao, 2008). China has the highest number of stroke cases in the world (Wang L. D. et al., 2022). Data from the Global burden of disease study (GBD, 2019 Diseases and Injuries Collaborators, 2020) indicates that stroke is the leading cause of death and disability among adults in China, with an ICH prevalence rate of 306 per 100,000 in 2019. Data from 1,672 public tertiary hospitals in the hospital quality monitoring system showed that ICH cases accounted for 14.2% of all stroke cases admitted for treatment in China in 2019 (Wang Y. J. et al., 2022). It is evident that ICH poses a serious threat to public health and impacts economic and social development. Currently, the primary treatment for ICH still focuses on symptomatic relief (Tang et al., 2018). Surgery may improve neurological recovery after ICH, but the presence of perihematomal edema post-surgery and potential secondary damage caused by the operation may limit its therapeutic effectiveness (Thompson et al., 2015).

Excitingly, TCM has accumulated a wealth of clinical experience in the treatment of ICH. With the approval of the National Medical Products Administration of China, Xingnaojing injection (XNJ), a derivative of Angong Niuhuang pill which have been used clinically for over 200 years, is a representative injectable drug in TCM used for the treatment of stroke, and it possesses the effects of clearing heat and detoxifying, cooling the blood and promoting circulation, as well as consciousness-restoring (Deng et al., 2010; Yue et al., 2019), Its formulation of Chinese botanical drugs comprises *Dryobalanops aromatica C.F.Gaertn* [Dipterocarpaceae; Borneolum], *Curcuma aromatica* Salisb. [Zingiberaceae; Curcumae Radix], *Gardenia jasminoides* J. Ellis [Rubiaceae; Gardeniae Fructus], *Moschus berezovskii Flerov, M. sifanicus* Przewalski, or *M. moschiferus* Linnaeus [Cervidae; Moschus], and it is produced using steam distillation to extract the water-soluble or volatile metabolites from the botanical drugs conveniently and effectively, resulting in an intravenous injection (Deng et al., 2010; Yue et al., 2019).

In China, XNJ is produced by three pharmaceutical companies (Wuxi Jiyu Shanhe Pharmaceutical Co., Ltd, Henan Tiandi Pharmaceutical Co., Ltd, and Dali Pharmaceutical Co., Ltd). The production processes and quality standards of all the three companies adhere to the National Drug Standards WS3-B-3353-98-2003 of China. In the preparation process, 30 g of Curcumae Radix and 30 g of Gardeniae Fructus are initially distilled with 1,500 mL of water, yielding 1,000 mL of distillate; subsequently, 7.5 g of Moschus and 250 mL of distilled water are introduced to the aforementioned distillate, followed by the collection of another 1,000 mL of distillate for later use. Next, 1 g of Borneolum and 8 g of polysorbate 80 are pulverized and combined with the distillate. Finally, 8 g of sodium chloride is incorporated, and the mixture is stirred, blended, left to settle overnight in a refrigerated environment, filtered, transferred to containers, and sterilized. Regarding the identified active components, borneol, which is traditionally utilized to monitor the quality of XNJ, should meet a minimum concentration of 0.7 g/L as stipulated by the drug standards set forth by the National Medical Products Administration of China (Pharmacopoeia Commission of the Ministry of Public Health of the People’s Republic of China, 1998; China Food and Drug Administration, 2003). Moreover, by using gas chromatography–mass spectrometry (GC-MS), high performance liquid chromatography (HPLC), network pharmacology, and molecular docking technology, researchers recently found that the representative active metabolites of XNJ also include muscone, camphor, eucarvone, isophorone, 4-methylene-isophorone, curcumenone, curcumenol, curdione, curzerenone, furanodienone, curcumol, germacrone, geniposide, etc. (Yang et al., 2016; Fang et al., 2017; Huang et al., 2017; Wu et al., 2021). A previous study has analyzed the 27 possible metabolites of XNJ, and found that among them, the camphor, borneol, and muscone account for more than 85% of the peak area of GC-MS (Zhang et al., 2004).

Numerous systematic reviews and meta-analyses have demonstrated the efficacy and safety of XNJ in the treatment of acute ICH (Peng et al., 2014; Wu et al., 2016; Yu et al., 2016; Xu et al., 2018; Ma et al., 2020; Wang et al., 2021). Many guidelines and consensus in China also recommend the use of XNJ for the emergency treatment of ICH (Gao, 2016; Ni et al., 2020; Gao and Zhao, 2023), but did not specify the recommendations and treatment advantages of XNJ application after the surgery of ICH. The results of one meta-analysis showed that for patients after ICH surgery, the addition of proprietary Chinese patent medicine (Naoxueshu oral liquid) had better clinical efficacy (Yu et al., 2023). Another network meta-analysis (Ren et al., 2022) found that compared with ICH patients who underwent surgery plus conventional western medicine (CWM) treatment, the addition of Chinese herbal injections on this basis could increase the total efficiency rate, lower National Institutes of Health Stroke Scale (NIHSS) scores, and improve Glasgow Coma Scale (GCS) scores, with good safety, and XNJ was ranked first in lowering NIHSS scores. Sadly, this study did not report mortality, perihematomal edema volume, or activities of daily living (ADL) ability, and did not specifically analyze and report the results of the traditional meta-analysis. Thus, there is currently a lack of systematic reviews and meta-analyses for the use of XNJ treatment in post-operative patients of ICH. Consequently, we used R 4.2.2 to invoke the meta package to perform a meta-analysis on the efficacy and safety of XNJ treatment after surgery of ICH, to provide evidence-based support for the application of it in this field.

## 2 Materials and methods

We performed this meta-analysis in strict accordance with the Preferred Reporting Items for Systematic Reviews and Meta-analyses (PRISMA) 2020 statement (Page et al., 2021). The protocol was already registered in PROSPERO (CRD42024503006).

### 2.1 Search strategy

Comprehensively searched the published RCTs included in PubMed, Cochrane Library, Embase, Web of Science, China National Knowledge Infrastructure (CNKI), VIP database, Wanfang Database, and SinoMed. Search period: from the inception of the databases to 31 January 2024. Languages: Chinese and English. Search method: using both Medical Subject Headings (MeSH) terms and free-text keywords. The search strategy was appropriately adjusted according to the individual features of each database. The detailed search strategies are provided in the Supplementary Material S1.

### 2.2 Eligibility criteria

#### 2.2.1 Inclusion criteria

(1) Study type: Randomized Controlled Trials (RCTs).
(2) Study subjects: Post-operative adult patients of ICH (as diagnosed by a clinician, or using any recognized diagnostic criteria). And surgical treatments including soft/hard channel puncture hematoma aspiration/fragmentation and drainage surgery, ventricular drainage surgery, neuroendoscopic hematoma evacuation surgery, craniotomy for hematoma removal, etc.
(3) Interventions: Both groups received CWM treatments (including hemostasis, dehydration, intracranial pressure reduction, blood pressure reduction, neural nutrition, cerebral cell activation, hyperbaric oxygen therapy, anti-infection, gastric acid suppression, etc.) as the foundation; the experimental group received additional intravenous injections of XNJ, with no restrictions on dosage or course of treatment.
(4) Outcome indicators: The study included at least one of the following outcomes: total efficiency rate; all-cause mortality; neurological impairment, assessed by NIHSS, European Stroke Scale (ESS), Chinese Stroke Scale (CSS), etc.; ability of ADL, assessed by Barthel Index (BI), modified Barthel Index (mBI), etc.; level of consciousness, assessed by GCS, etc.; volume of intracerebral hematoma; volume of perihematomal edema; levels of inflammatory indicator TNF- α, and safety indicators (including adverse drug reactions and incidence of complications).

#### 2.2.2 Exclusion criteria

(1) Study that was grouped by incorrect random methods such as the order of admission or treatment method.
(2) Intervention measures include other therapies, in addition to XNJ (intravenous injection) and CWM treatment.
(3) Study with statistical errors where the data cannot be aggregated.
(4) For the same literature published repeatedly, one with complete data was reserved, and the rest were excluded.
(5) For studies with completely duplicated data but different authors, the latest published studies were excluded.

### 2.3 Study selection and data extraction

Two researchers independently screened and extracted information from the literature according to the inclusion and exclusion criteria set forth in the study. In cases where there was disagreement, a discussion was initiated; if disagreements persisted, a third party would consider the different viewpoints and make a decision. The extracted information by preformulated data collection form included the first author, publication year, sample size, gender, age, interventions, duration of treatment, outcome indicators, and their respective data.

### 2.4 Assessment of risk of bias

The quality of the studies was assessed using the Risk of Bias assessment tool (ROB2.0) in the Cochrane Handbook (v6.4) (Higgins et al., 2023). The assessment was cross-checked by two researchers, and any discrepancies were resolved through discussion. If consensus could not be reached, a third researcher was consulted to make a decision. The assessment covered the following domains: randomization process, deviations from intended interventions, missing outcome data, measurement of the outcome, selection of the reported result, and overall risk of bias. Each domain encompassed one to seven specific questions. The results were classified as “low risk of bias”, “some concerns” and “high risk of bias".

### 2.5 Statistical analysis

Meta-analysis was carried out using R 4.2.2. The effect size for dichotomous variables was expressed as relative risks (RR) with their 95% confidence intervals (CI), and a continuity correction of 0.5 was added to each side when there were studies with 0 cells.

For continuous variables, when the outcomes were in the same unit, the weighted mean difference (WMD) with its 95% CI was calculated using the noStandard method; when the units varied or the scales different for the same outcome in different studies, the standardized mean difference (SMD) with its 95% CI was computed using Hedges’ method.

Before combining effect sizes, an assessment ( $\chi^{2}$ and $I^{2}$ test) for heterogeneity was conducted, and a fixed-effect model was used if *p* ≥ 0.10 and I^2^ < 50%; otherwise, a random-effects model was employed, and subgroup analyses were undertaken to explore the sources of heterogeneity. Sensitivity analysis was also used to investigate the stability of study results and the sources of heterogeneity. Potential publication bias was explored using Begg’s test and Egger’s test. It was considered statistically significant when *p* < 0.05.

### 2.6 Trial sequential analysis (TSA) and quality of evidence

Trial sequential analysis (TSA) was performed using TSA 0.9.5.10 Beta (Thorlund et al., 2017). And calculated the Required Information Size (RIS) to determine the possibility of false negative results. The quality of evidence was graded using the web-based development tool of GRADEpro GDT (https://www.gradepro.org/). Based on the GRADE methodology applied in systematic reviews (Guyatt et al., 2011), three upgrading factors (potential confounders, dose-response relationship, large magnitude of effect) and five downgrading factors (publication bias, indirectness, heterogeneity, imprecision, and risk of bias) were thoroughly considered to classify the quality of evidence into four levels: very low, low, moderate, and high. If more than one downgrading factor was present, the quality of evidence would be downgraded, resulting in the formation of an evidence profile.

## 3 Results

### 3.1 Literature search

**FIGURE 1:**
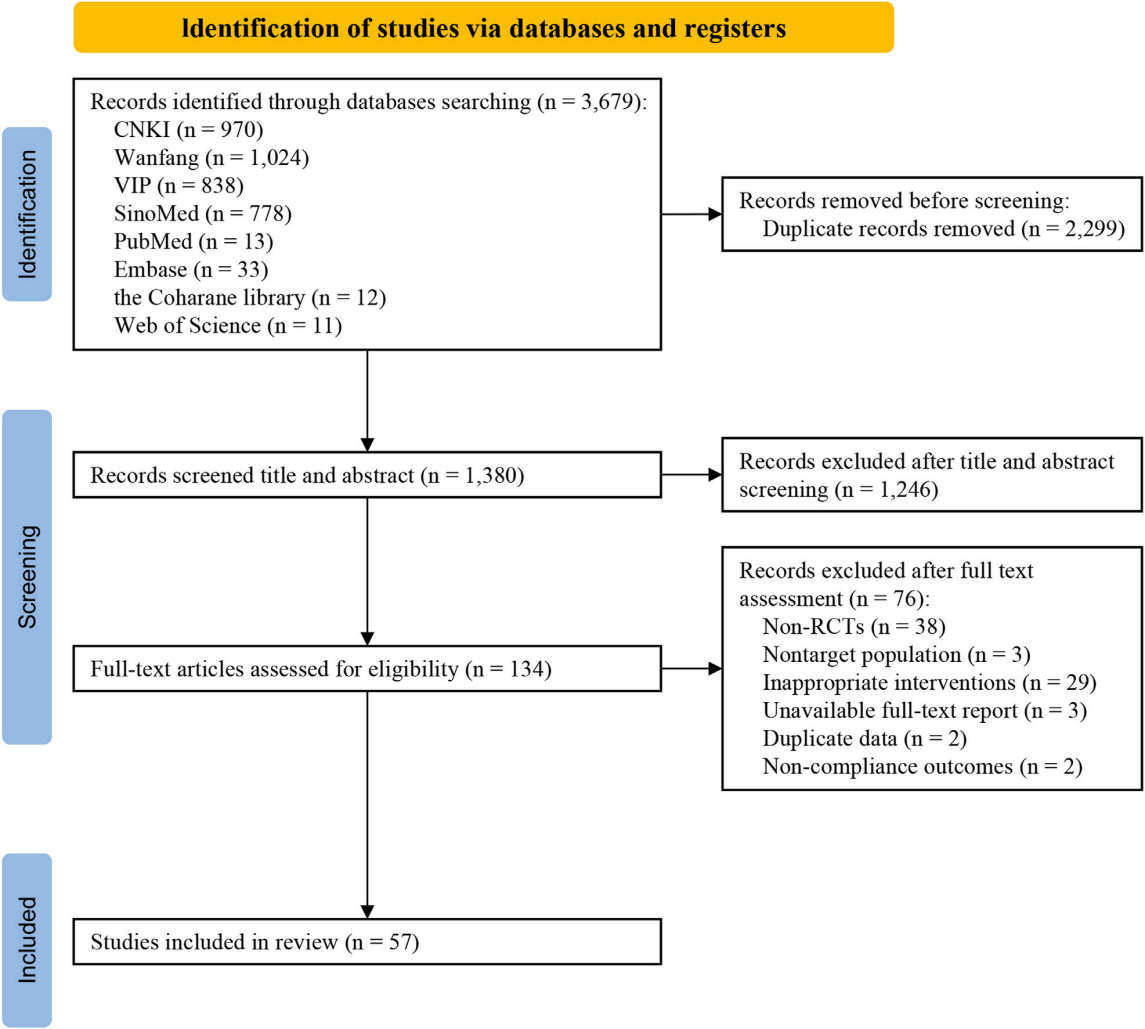
Flow chart of the study selection process.

In this study, a total of 3,679 studies were retrieved, including 3,610 Chinese studies and 69 English studies. The retrieved study citations were imported into NoteExpress v3.5, and a total of 57 studies (Wang et al., 2001; Lin et al., 2003; Wu and Han, 2004; Huang, 2005; Tong et al., 2006; Lin, 2009; Nie, 2010; Li et al., 2011; Lu and Tang, 2011; Wu et al., 2011; Yang et al., 2011; Zhao, 2011; Li, 2012; Li et al., 2012; Li and Zhou, 2012; Jin, 2013; Shi et al., 2013; Sun and Zhong, 2013; Tao et al., 2013; Xin, 2013; Guo and Wen, 2014; Huang and Guo, 2014; Zhou, 2014; Chen and Li, 2015; Cheng, 2015; Dai et al., 2015; Guo, 2015; He et al., 2015; Tang, 2015; Xu and Lv, 2015; Zhang et al., 2015; Zhou, 2015; Lian, 2016; Liu et al., 2016; Ren, 2016; Tong et al., 2016; Xia et al., 2016; Zhang et al., 2016; Gu and Zhang, 2017; Shuang et al., 2017; Zhou and Sun, 2017; Cheng, 2018; Jiang and Xiao, 2018; Jin and Wang, 2018; Li et al., 2018; Li, 2019; Wang, 2019; You, 2019; Zhang, 2019; Liang and Qin, 2020; Shu, 2020; Xu, 2020; Chen, 2021; Deng et al., 2021; Xiao and Wu, 2021; Sun et al., 2022; Hao et al., 2024) were ultimately included after screening (Figure 1).

### 3.2 Characteristics of the included studies

This study incorporated a total of 57 RCTs, involving 4,852 patients, with 2,445 in the integration of XNJ and CWM therapy group (experimental group) and 2,407 in the CWM treatment group (control group). All studies were conducted in China and were single-center RCTs. The sample sizes ranged from 29 to 183. All RCTs were based on surgery and CWM treatment, with one group receiving additional XNJ (Table 1).

**TABLE 1:** Characteristics of the included trials.

Study	Type of ICH	Sample size		Sex (M/F)		Age (range/mean) (years)		Intervention (T)		Time of surgery (range/mean)		Duration	Outcomes
		T	C	T	C	T	C	XNJ	Surgery	T	C		
Hao et al. (2024)	ICH	50	50	25/25	24/26	45-80/62.51 ± 7.51	45-79/62.48 ± 7.04	20 mL qd	Unclear	-	-	2w	③⑤⑨
Sun et al. (2022)	HICH	61	61	30/31	31/30	67-81	66-82	30 mL qd	NES	2–18 h	3–19 h	1w	①③④⑥⑧⑨
Xiao and Wu (2021)	ICH	30	30	15/15	14/16	≥60/65.87 ± 3.80	≥60/66.47 ± 3.55	10–20 mL qd	Mix	-		2w	①③④⑧⑨
Deng et al. (2021)	HICH	50	50	30/20	33/17	45-69/50.72 ± 3.00	46-69/50.98 ± 3.33	20 mL qd	SCPADS	≤24 h		2w	①
Chen (2021)	ICH	29	29	12/17	16/13	45-70/57.1 ± 5.6	42-71/57.3 ± 5.4	20 mL qd	Unclear	-		2w	③④⑥
Liang and Qin (2020)	HICH	24	24	14/10	15/9	56-78/65.38 ± 4.26	58-75/65.80 ± 4.15	20 mL qd	HCPADS	≤36 h		2w	①③⑤
Xu (2020)	HICH	55	55	33/22	35/20	73.79 ± 7.46	75.57 ± 8.52	20 mL qd	HCPADS	≤6 h		1w	③⑥
Shu (2020)	HICH	41	41	27/14	28/13	51-74/62.53 ± 1.25	51-75/62.58 ± 1.21	20 mL qd	HCPADS	<48/14.17 ± 5.36 h	<48/14.20 ± 5.31 h	30d	①③⑤
Zhang (2019)	ICH	40	40	18/12	29/11	38-67/51.95 ± 9.93	30-70/51.42 ± 7.46	10–20 mL qd	HCPADS	-		2w	①⑦
Li (2019)	HICH	45	45	48/42		51-72/64.2 ± 6.3		30 mL qd	SCPADS	5–7 h		2w	⑤
Wang (2019)	HICH	59	59	32/27	33/26	57.24 ± 8.02	58.09 ± 7.65	20 mL qd	HCPADS	≤48/14.02 ± 8.95 h	≤48/13.97 ± 9.02 h	4w	①③⑧⑨
You (2019)	HICH	37	37	25/12	24/13	42-70/49.2 ± 5.3	39-68/48.8 ± 5.9	10–20 mL bid	Unclear	-		20d	①②⑤⑨
Li et al. (2018)	ICH	51	51	30/21	29/22	42-74/54.8 ± 8.4	41-76/52.7 ± 7.1	20 mL qd	SCPADS	≤72 h		6w	③④
Jiang and Xiao (2018)	HICH	25	25	16/9	14/11	55-74/66.5 ± 5.2	55-75/66.1 ± 5.3	10–20 mL qd	SCPADS	-		2w	①⑤⑥
Cheng (2018)	HICH	20	20	12/8	11/9	28-66/56.3 ± 6.2	29-67/56.8 ± 6.1	20 mL qd	SCPADS	1.0-21.7/8.7 ± 2.5 h	0.8-21.3/8.8 ± 2.4 h	2w	①③⑤⑦
Jin and Wang (2018)	HICH	42	42	28/14	29/13	62.3 ± 5.1	61.6 ± 4.8	20 mL qd	HCPADS	-		4w	①②③⑧⑨
Shuang et al. (2017)	HICH	54	54	56/52		43-74/63.1 ± 10.2		20 mL qd	NES	1-36/10.3 ± 3.4 h		4w	①③⑧
Gu and Zhang (2017)	HICH	44	44	26/18	24/20	24-78/56.01 ± 6.05	25-80/55.68 ± 5.98	20 mL qd	SCPADS	-		2w	①③⑥⑨
Zhou and Sun (2017)	HICH	50	50	26/24	28/22	38-72/51.8 ± 3.2	36-71/46.8 ± 2.5	30 mL qd	Mix	-		30d	①⑨
Zhang et al. (2016)	HICH	37	37	27/10	25/12	52-70/61.5 ± 4.9	53-72/61.8 ± 4.6	20 mL qd	HCPFAS	6-20/12.5 ± 4.1 h	5-19/12.8 ± 3.7 h	2w	①②⑧
Xia et al. (2016)	HICH	44	44	29/15	30/14	35-78/60.1 ± 3.5	38-76/59.6 ± 3.6	40 mL qd	HCPFAS	-		2w	①③⑤⑥
Ren (2016)	HICH	53	53	65/41		35-77/63.6 ± 5.6		4 mL qd	HCPADS	5.3 ± 1.7 h		2w	③
Liu et al. (2016)	ICH	49	49	65/53		52-78/65.8 ± 4.6		20–30 mL qd	Unclear	-		4w	①②③
Lian (2016)	ICH	80	80	47/33	48/32	44-80/58 ± 6	45-80/54 ± 8	30 mL qd	HCPADS	12–72 h		3w	③④⑨
Tong et al. (2016)	HICH	68	72	38/30	36/36	57.12 ± 5.42	58.38 ± 6.03	30 mL qd	Mix	-		15d	①⑨
Zhang et al. (2015)	HICH	50	50	54/46		61.2 ± 7.5		30 mL qd	SCPADS	5–8 h		2w	②③
Xu and Lv (2015)	HICH	52	52	29/23	27/25	44-72/59.12 ± 10.36	41-76/58.69 ± 11.02	20 mL qd	Mix	-		1 m	①
Tang (2015)	ICH	50	50	30/20	29/21	53-76/65.2	51-77/64.9	30 mL qd	HCPADS	-		-	①③④
He et al. (2015)	HICH	40	40	23/17	25/15	52-75/62.4 ± 8.0	50-71/61.8 ± 7.2	20 mL qd	HCPFAS	6-47/15.2 ± 11.9 h	4-41/14.8 ± 10.2 h	2w	①③
Guo (2015)	HICH	40	40	-		-		20 mL qd	PADS	-		3w	①
Dai et al. (2015)	HICH	30	30	22/8	24/6	29-61/51.95 ± 7.93	28-68/52.83 ± 7.52	20 mL qd	HCPADS	-		2w	①⑦
Cheng (2015)	HICH	60	59	37/23	36/24	49-76/58.92 ± 7.48	50-78/60.05 ± 8.36	20 mL bid	Unclear	-		2w	①②③⑤⑨
Chen and Li (2015)	HICH	46	44	26/20	23/21	52-76/65.2 ± 6.5		30 mL qd	SCPADS	-		2w	①②③⑤
Zhou (2015)	HICH	97	86	53/44	51/35	42-75	40-75	20 mL qd	HCPFAS	1–36 h	0.5–34 h	2w	①
Zhou (2014)	HICH	48	48	25/23	26/22	38-78/58.14 ± 7.98	39-80/60.02 ± 7.05	20 mL bid	HCPFAS	48 h		2w	①
Guo and Wen (2014)	ICH	61	61	41/20	41/20	52-78/65.23 ± 3.49	51-79/65.52 ± 3.51	30 mL qd	PADS	9-58/17.63 ± 2.94 h	8-63/17.72 ± 2.89 h	2w	①③④
Huang and Guo (2014)	HICH	52	50	68/34		47-79/57.1 ± 6.2		20 mL bid	NES	-		15d	①②③
Xin (2013)	HICH	42	42	22/20	24/18	41-78/58.6 ± 8.4	43-79/57.2 ± 9.4	40 mL qd	HCPFAS	4-15/8.5 ± 4.4 h	3-17/8.4 ± 3.9 h	2w	①②③
Tao et al. (2013)	HICH	42	38	23/19	18/20	49-64/55.4 ± 4.6	56.7 ± 5.4	30 mL qd	SCPADS	-		2w	①③⑦
Sun and Zhong (2013)	HICH	30	30	-		-		20 mL qd	HCPADS	-		2w	①
Shi et al. (2013)	HICH	40	40	48/32		17-82/56 ± 18		40 mL qd	HCPFAS	-		2w	①②⑧
Jin (2013)	ICH	24	26	13/11	13/13	40-75/59.7 ± 8.3	42-78/57.4 ± 9.1	40 mL qd	Craniotomy	4.5-14/8.2 ± 4.6 h	2.5-16/8.1 ± 3.8 h	2w	①
Li and Zhou (2012)	HICH	21	20	11/10	10/10	56-75/65 ± 4.8	56-74/65.2 ± 4.9	20–30 mL qd	HCPFAS	4-21/9.6–1.26 h		2w	①②④
Li (2012)	HICH	25	20	15/10	11/9	35-79/53.2 ± 8.3	34-81/53.7 ± 5.9	20 mL qd	SCPADS	6–72 h		1 m	①②⑨
Li et al. (2012)	ICH	28	26	17/11	17/9	49-72/57.60 ± 14.9	45-70/2.40 ± 11.7	20 mL qd	HCPFAS	6–48 h		1w	③④⑥
Zhao (2011)	ICH	45	45	30/15	31/14	50-75/45-77	45-77	20–30 mL qd	Unclear	-		2w	①②③
Yang et al. (2011)	HICH	30	30	17/13	16/14	46-78/62.4	44-75/61.2	20 mL qd	Mix	5h-5d	5.5h-6d	2w	①②⑨
Wu et al. (2011)	HICH	20	20	-		-		20 mL qd	Unclear	-		-	①②
Li et al. (2011)	ICH	58	57	33/25	34/23	53.1 ± 8.4	51.8 ± 7.8	20 mL qd	HCPADS	14.9 ± 6.6d	15.5 ± 4.9d	2w	①④
Lu and Tang (2011)	HICH	54	54	30/24	31/23	36-68/58.7	35-69/59.1	20 mL qd	Unclear	-		2w	①②③
Nie (2010)	HICH	20	20	14/6	13/7	45-75		30 mL qd	Craniotomy	≤24 h		2w	①④⑨
Lin (2009)	HICH	24	22	18/6	16/6	56.25 ± 4.19	57.73 ± 4.47	40 mL qd	SCPADS	6.17 ± 1.19 h	5.34 ± 0.89 h	2w	①④⑤
Tong et al. (2006)	HICH	42	38	24/18	22/16	50-82/64.2 ± 4.9	53-78/66.1 ± 3.8	20 mL qd	HCPFAS	5-25/10.6 ± 1.63 h	4-21/9.6 ± 1.26 h	2w	①②⑨
Huang (2005)	HICH	14	15	10/4	11/4	51-78/64.5	53-79/65.1	10 mL qd	Unclear	6-19/12.6 h	7-20/11.2 h	2w	③⑦
Wu and Han (2004)	HICH	25	20	14/11	12/8	40-82		20 mL qd	HCPFAS	6–72 h		-	①②⑨
Lin et al. (2003)	ICH	37	32	21/16	23/9	51-74/59.3	54-75/61.2	1.0 mL/kg qd	Mix	≤24 h		2w	①⑤⑨
Wang et al. (2001)	ICH	60	60	37/23	39/21	37-71/60.2	36-70/59.2	20 mL qd	Mix	0.5–72 h	0.5–72 h	4w	①③

Note: NES, neuroendoscopic hematoma evacuation surgery; Mix, the included patients underwent different surgical measures; SCPADS, soft channel puncture hematoma aspiration and drainage surgery; HCPADS, hard channel puncture hematoma aspiration and drainage surgery; HCPFAS, hard channel puncture hematoma fragmentation and aspiration surgery; PADS, puncture hematoma aspiration and drainage surgery; Unclear, only mentioning minimally invasive surgery; ①, total efficiency rate; ②, all-cause mortality rate; ③, neurological impairment; ④, level of consciousness; ⑤, Activities of daily living (ADL); ⑥, volume of intracerebral hematoma; ⑦, volume of perihematomal edema; ⑧levels of TNF- α, ⑨, safety indicators.

### 3.3 Risk of bias assessment

**FIGURE 2:**
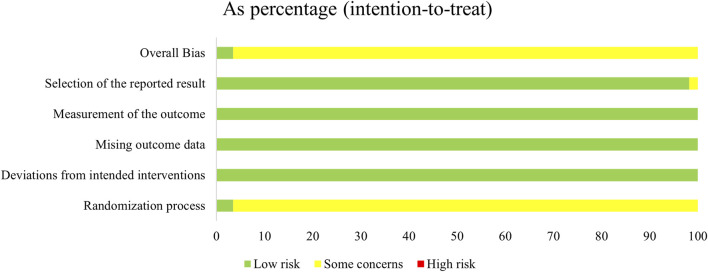
Risk of bias graph.

Regarding the “randomization process”, all studies reported comparability of baseline data between the two groups, and with 24 studies (Lin et al., 2003; Lin, 2009; Li, 2012; Tao et al., 2013; Guo and Wen, 2014; Chen and Li, 2015; Dai et al., 2015; Xu and Lv, 2015; Lian, 2016; Ren, 2016; Xia et al., 2016; Zhang et al., 2016; Gu and Zhang, 2017; Cheng, 2018; Jiang and Xiao, 2018; Jin and Wang, 2018; Li et al., 2018; Li, 2019; Wang, 2019; You, 2019; Liang and Qin, 2020; Chen, 2021; Hao et al., 2024) reported specific and correct randomization methods. But only two studies (Lin, 2009; Nie, 2010) of them assessed as “low risk of bias” because they explicitly mentioned the use of opaque envelopes to conceal allocations. In contrast, the remaining studies were assessed as “some concerns” due to the absence of specific randomization strategies or conceal allocations mentioned. For the “deviations from the intended interventions”, we assessed it as “low risk of bias”. Although only one study (Liu et al., 2016) used a double-blind design, and two studies (Jin, 2013; Tong et al., 2016) administered a placebo treatment (intravenous saline injection) to the control group. However, 14 studies (Lin, 2009; Nie, 2010; Li et al., 2011; Li et al., 2012; Li and Zhou, 2012; Guo and Wen, 2014; He et al., 2015; Tang, 2015; Lian, 2016; Li et al., 2018; Zhang, 2019; Chen, 2021; Xiao and Wu, 2021; Sun et al., 2022) mentioned that all included patients had varying degrees of consciousness impairment, we expect these patients likely did not know which treatment measures they were receiving. There were no instances of patients switching groups due to awareness or unawareness of their treatment modalities. All studies used an intention-to-treat analysis (ITT) to estimate the effects of allocated intervention measures, and were assessed to be at low risk. In addition, we assessed the “missing outcome data” as “low risk” due to no loss to follow-up was reported, or negligible losses to follow-up was founded. The “outcome measurements” were assessed as “low risk” because the criteria for evaluating the outcome indicators between the two groups were reasonable and consistent in all studies. Although they did not mention whether blinding was implemented for the outcome assessors, it is still unclear whether this would affect the judgment of the results, as the outcome assessors’ potential preference bias towards the two treatment measures is unknown. Apart from one study (Huang, 2005) (assessed as 'some concerns’ due to the safety situation reported only for the experimental group), we assessed all remaining studies as having a “low risk of bias” in the case of “selective reporting” because all of them had clear outcome indicators and comprehensively reported results whether they were statistically significant or not (Figure 2; Supplementary Figure S1).

### 3.4 Primary outcomes

#### 3.4.1 Total efficiency rate

**FIGURE 3:**
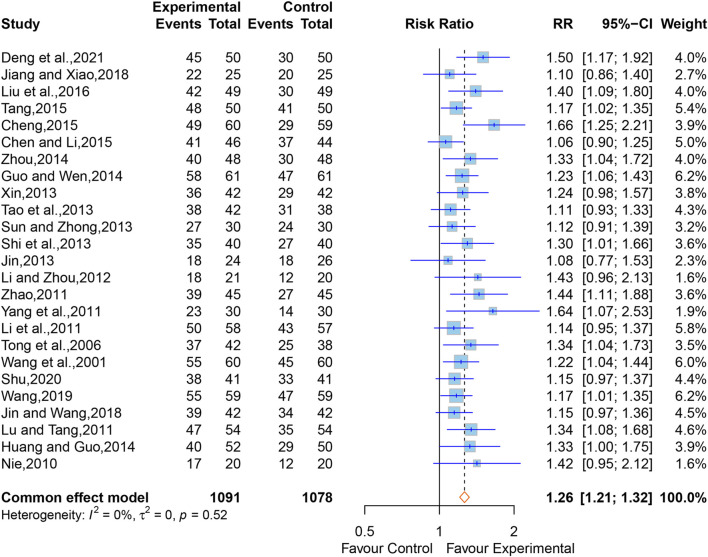
Forest plot for the effect of Xingnaojing on the total efficiency rate.

A total of 47 studies (Wang et al., 2001; Lin et al., 2003; Wu and Han, 2004; Tong et al., 2006; Lin, 2009; Nie, 2010; Li et al., 2011; Lu and Tang, 2011; Wu et al., 2011; Yang et al., 2011; Zhao, 2011; Li, 2012; Li and Zhou, 2012; Jin, 2013; Shi et al., 2013; Sun and Zhong, 2013; Xin, 2013; Guo and Wen, 2014; Huang and Guo, 2014; Zhou, 2014; Chen and Li, 2015; Cheng, 2015; Dai et al., 2015; Guo, 2015; He et al., 2015; Tang, 2015; Xu and Lv, 2015; Zhou, 2015; Liu et al., 2016; Tong et al., 2016; Xia et al., 2016; Zhang et al., 2016; Gu and Zhang, 2017; Shuang et al., 2017; Zhou and Sun, 2017; Cheng, 2018; Jiang and Xiao, 2018; Jin and Wang, 2018; Wang, 2019; You, 2019; Zhang, 2019; Liang and Qin, 2020; Shu, 2020; Deng et al., 2021; Xiao and Wu, 2021; Sun et al., 2022) comprising 3,943 participants reported the total efficiency rate. We first conducted a meta-analysis on 25 of the studies (Wang et al., 2001; Tong et al., 2006; Nie, 2010; Li et al., 2011; Lu and Tang, 2011; Yang et al., 2011; Zhao, 2011; Li and Zhou, 2012; Jin, 2013; Shi et al., 2013; Sun and Zhong, 2013; Tao et al., 2013; Xin, 2013; Guo and Wen, 2014; Huang and Guo, 2014; Zhou, 2014; Chen and Li, 2015; Cheng, 2015; Tang, 2015; Liu et al., 2016; Jiang and Xiao, 2018; Jin and Wang, 2018; Wang, 2019; Shu, 2020; Deng et al., 2021) which used “18% reduction in post-treatment neurological impairment scales scores” as the criterion for total efficiency rate. A fixed-effect model was used due to the low heterogeneity (I^2^ = 0%, *p* = 0.52), and the pooled data showed that XNJ significantly improved the total efficiency rate (RR = 1.26; 95% CI [1.21 to 1.32]; *p* < 0.0001) (Figure 3). Sensitivity analysis clarified that the combined effect size was stable (Supplementary Figure S2).

Considering that different studies have adopted various methods of evaluating therapeutic efficacy, further analyses were then performed, respectively, by different efficacy evaluation criteria. The results indicated that there were no significant differences in treatment efficacy among them (*p =* 0.58) (Supplementary Figure S12).

### 3.5 Secondary outcomes

#### 3.5.1 All-cause mortality

**FIGURE 4:**
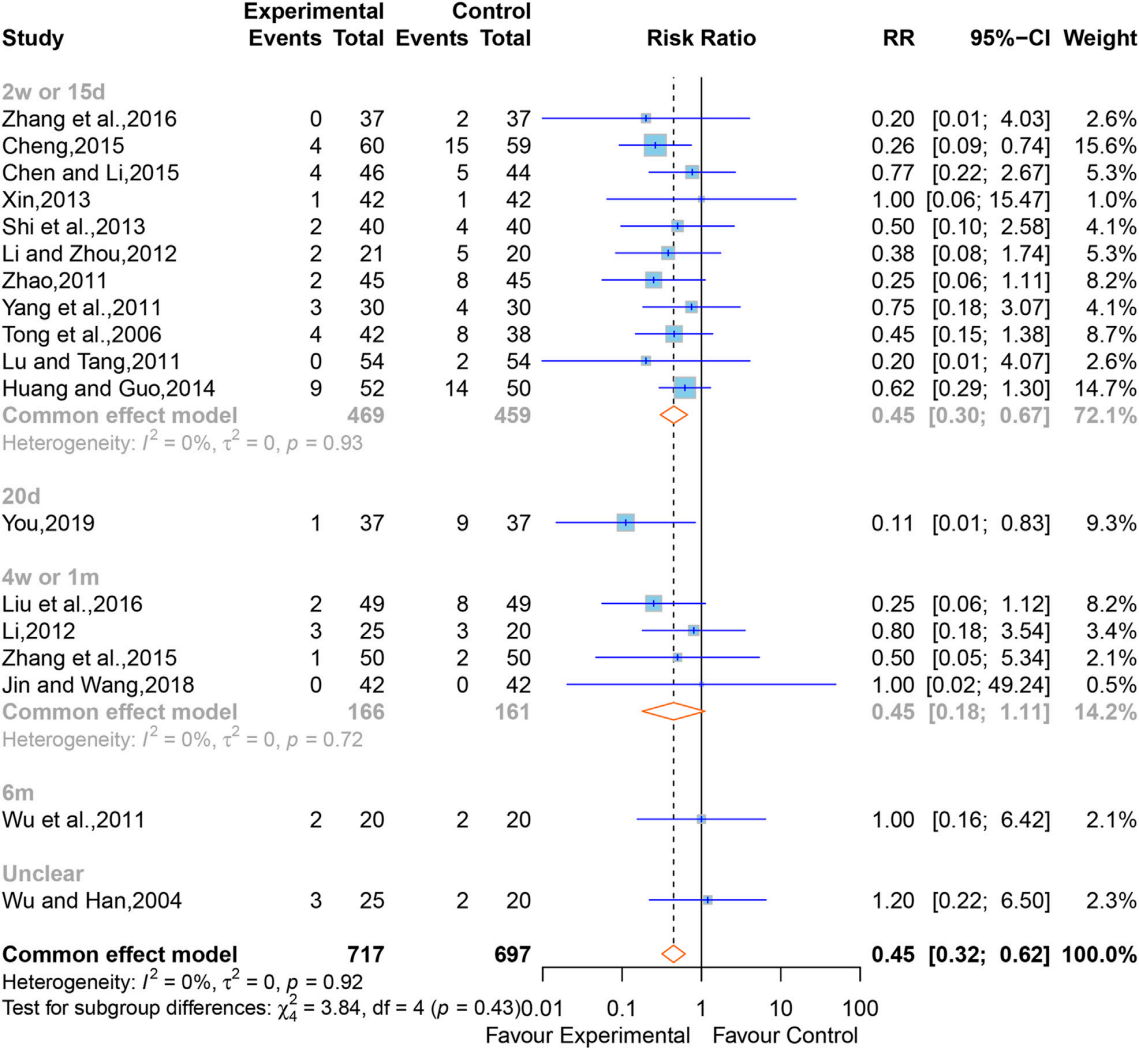
Forest plot for the effect of Xingnaojing on the all-cause mortality.

In all, 18 studies (Wu and Han, 2004; Tong et al., 2006; Lu and Tang, 2011; Wu et al., 2011; Yang et al., 2011; Zhao, 2011; Li, 2012; Li and Zhou, 2012; Shi et al., 2013; Xin, 2013; Huang and Guo, 2014; Chen and Li, 2015; Cheng, 2015; Zhang et al., 2015; Liu et al., 2016; Zhang et al., 2016; Jin and Wang, 2018; You, 2019) containing 1,414 cases reported the all-cause mortality. The overall effect of meta-analysis indicated that XNJ reduced the all-cause mortality (RR = 0.45; 95% CI [0.32 to 0.62]; *p* < 0.0001), and a fixed-effects model was applied due to the low heterogeneity (I^2^ = 0%, *p* = 0.92) (Figure 4). Sensitivity analysis indicated that the combined effect size was stable (Supplementary Figure S3).

Subgroup analyses were conducted according to the time point of observation. The outcomes clarified that, compared to the control group, XNJ significantly reduced all-cause mortality after 2 weeks or 15 days of starting treatment. However, at the periods of 4 weeks or 1 month, 6 months, and unclear time for observing the all-cause mortality, the result of the XNJ group was no significant compared with that of the control group. Additionally, for the 20 days, 6 months, and unclear groups, there was only one study each (2w or 15d, RR = 0.45; 95% CI [0.30 to 0.67]; 20d, RR = 0.11; 95% CI [0.01 to 0.83]; 4w or 1m, RR = 0.45; 95% CI [0.18 to 1.11]; 6m, RR = 1.00; 95% CI [0.16 to 6.42]; Unclear, RR = 1.20; 95% CI [0.22 to 6.50]) (Figure 4).

#### 3.5.2 Neurological impairment

Regarding neurological impairment, 15 studies (Li et al., 2012; Chen and Li, 2015; Zhang et al., 2015; Lian, 2016; Ren, 2016; Shuang et al., 2017; Cheng, 2018; Jin and Wang, 2018; Li et al., 2018; Liang and Qin, 2020; Shu, 2020; Xu, 2020; Xiao and Wu, 2021; Sun et al., 2022; Hao et al., 2024) containing 1,414 cases reported the grading by NIHSS, seven studies (Wang et al., 2001; Huang, 2005; Zhao, 2011; Li, 2012; Tao et al., 2013; Huang and Guo, 2014; He et al., 2015; Liu et al., 2016) containing 1,414 cases reported the grading by CSS, two studies (Cheng, 2015; Xia et al., 2016) containing 1,414 cases reported the grading by ESS, and seven studies (Lu and Tang, 2011; Xin, 2013; Guo and Wen, 2014; Tang, 2015; Gu and Zhang, 2017; Wang, 2019; Chen, 2021) did not specify the evaluation scale used.

**FIGURE 5:**
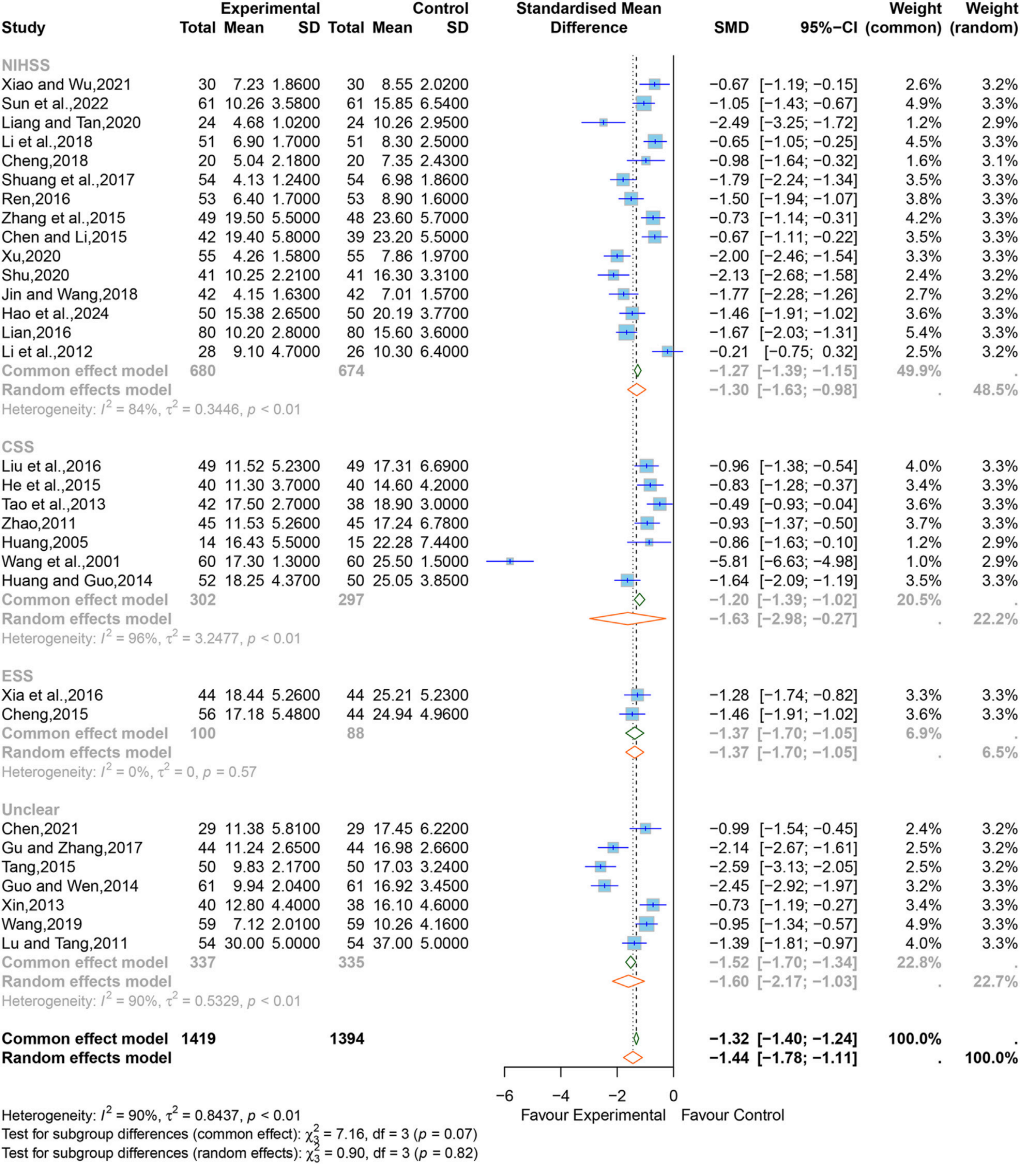
Forest plot for the effect of Xingnaojing on neurological impairment.

The SMD was chosen to standardize the effect sizes across studies to counteract the total score differences caused by the use of different scales among the studies. Due to significant heterogeneity among them (I^2^ = 90%, *p <* 0.01), a random-effects model was applied. The results indicated that XNJ significantly improved neurological impairment (SMD = −1.44; 95% CI [−1.78 to −1.11]; *p* < 0.0001) (Figure 5). Sensitivity analysis showed that the results of the combined effect size are stable (Supplementary Figure S4).

Based on the subgroup analyses of different evaluation scales, it can be seen that the heterogeneity of the ESS group was significantly reduced (I^2^ = 0%, *p =* 0.57), but the CSS, the NIHSS, and the unclear group were relatively high (NIHSS, I^2^ = 84%, *p* < 0.01; CSS, I^2^ = 96%, *p* < 0.01; unclear, I^2^ = 90%, *p* < 0.01) (Figure 5). The rest of the subgroup analyses are listed in Supplementary Table S1. The overall effect combined by MD showed that there was still statistical significance in each subgroup, which further verified the reliability of the results (NIHSS, MD = −3.56; 95% CI [−4.40 to −2.72]; CSS, MD = −5.26; 95% CI [−7.18 to −3.35]; ESS, MD = −7.30; 95% CI [−8.80 to −5.80]; unclear, MD = −5.65; 95% CI [−6.99 to −4.32]) (Supplementary Figure S13).

#### 3.5.3 Consciousness

**FIGURE 6:**
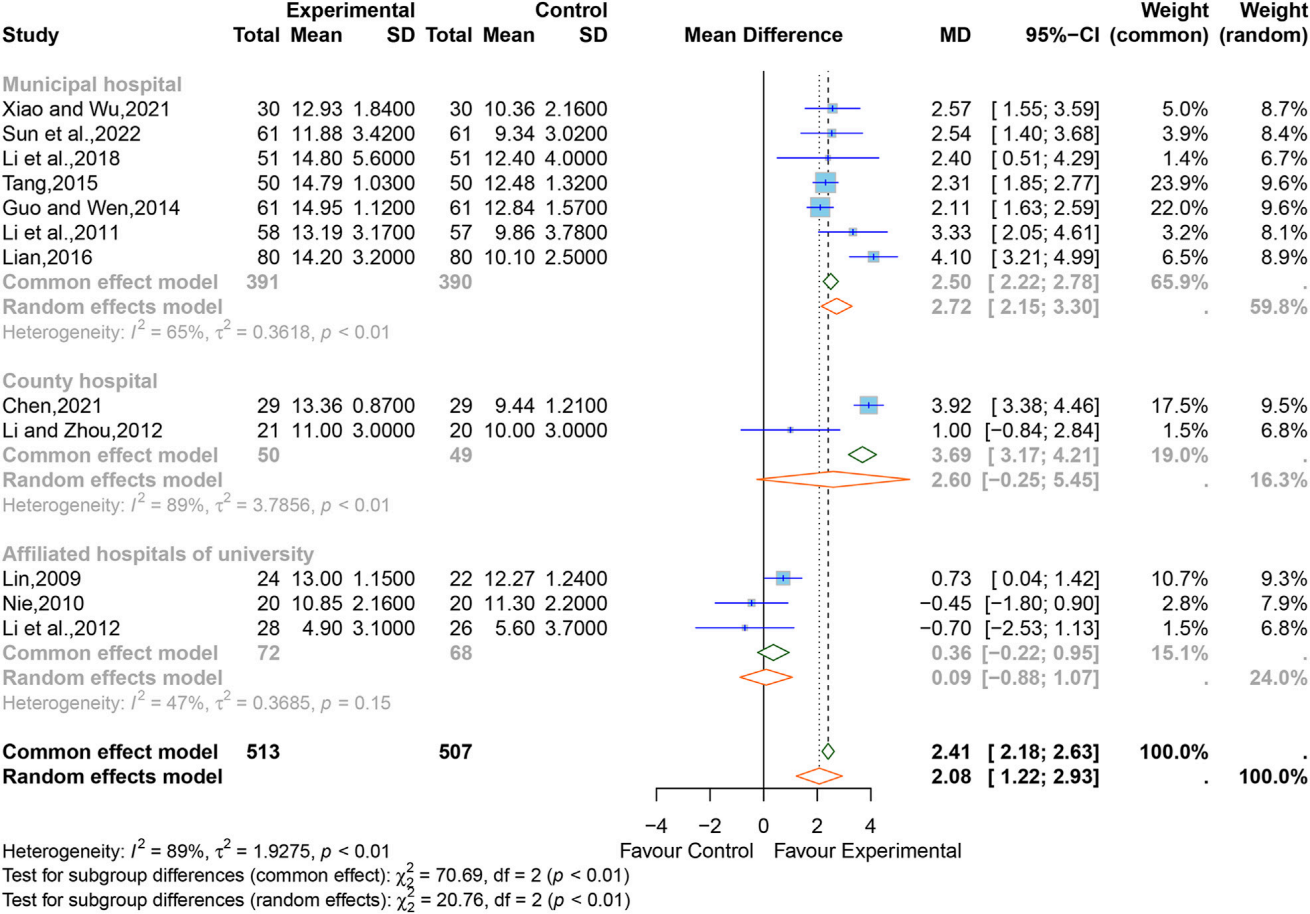
Forest plot for the effect of Xingnaojing on the Glasgow coma scale.

Of all studies, 12 studies (Lin, 2009; Nie, 2010; Li et al., 2011; Li et al., 2012; Li and Zhou, 2012; Guo and Wen, 2014; Tang, 2015; Lian, 2016; Li et al., 2018; Chen, 2021; Xiao and Wu, 2021; Sun et al., 2022) comprising 1,020 participants reported the state of consciousness of patients after treatment, all evaluated using the GCS. In view of the significant heterogeneity between the studies (I^2^ = 89%, *p* < 0.01), thus, a random-effects model was used. The result showed that XNJ significantly improved the GCS scores (MD = 2.08, 95% CI [1.22 to 2.93], *p* < 0.0001) (Figure 6). Sensitivity analysis indicated that the result was stable (Supplementary Figure S5).

The results of the subgroup analyses based on “hospital” demonstrated that the heterogeneity of the “affiliated hospital of university” group was significantly reduced (I^2^ = 47%, *p* = 0.15), the heterogeneity within the other subgroups still remained high (county hospital, I^2^ = 89%, *p <* 0.01; municipal hospital, I^2^ = 65%, *p <* 0.01), and the results for both the “affiliated hospital of university” and “county hospital” groups indicated that the GCS scores of the XNJ group was non-significant compared with that of the control group (affiliated hospital of university, MD = 0.36, 95% CI [-0.22 to 0.95]; county hospital, MD = 2.60, 95% CI [-0.25 to 5.45]). However, the results from the “municipal hospital” group showed that there is a statistically significant difference between the two groups (MD = 2.72, 95% CI [2.15 to 3.30]). The rest of the subgroup analyses are listed in Supplementary Table S2.

#### 3.5.4 Activities of daily living

After treatment, three studies (Chen and Li, 2015; Cheng, 2015; Xia et al., 2016) containing 269 cases used BI scores to evaluate ADL, two studies (Lin, 2009; Li, 2019) containing 136 cases used mBI, two studies (You, 2019; Hao et al., 2024) containing 174 cases used other scales (SS-QOL, QLQ-C30), and five studies (Lin et al., 2003; Cheng, 2018; Jiang and Xiao, 2018; Liang and Qin, 2020; Shu, 2020) containing 289 cases did not specify the type of scale used.

**FIGURE 7:**
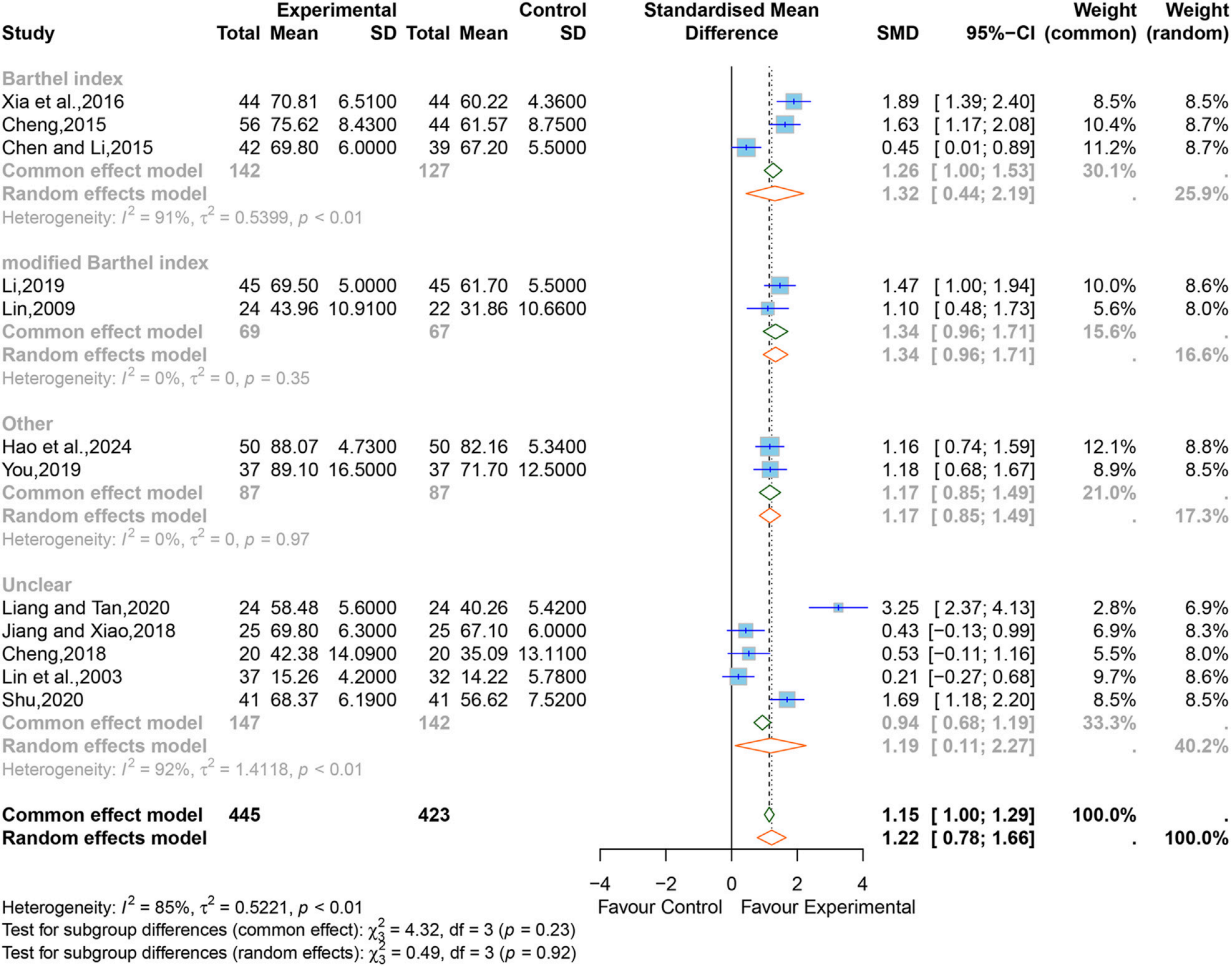
Forest plot for the effect of Xingnaojing on the activities of daily living.

There was high heterogeneity between the studies (I^2^ = 85%, *p* < 0.01), therefore a random-effects model was used with SMD as a summary statistic. The results showed that the difference was statistically significant (SMD = 1.22; 95% CI [0.78 to 1.66]; *p* < 0.0001) (Figure 7). Sensitivity analysis showed that the overall effect was stable (Supplementary Figure S6).

We performed subgroup analyses, respectively, by the different evaluation scales, and the results indicated that the heterogeneity of the “mBI” and “other scales" groups were significantly reduced (mBI, I^2^ = 0%, *p* = 0.35; other scales, I^2^ = 0%, *p* = 0.97), the heterogeneity within the other subgroups still remained high (BI, I^2^ = 91%, *p <* 0.01; Unclear, I^2^ = 92%, *p <* 0.01). The rest of the subgroup analyses are listed in Supplementary Table S3. The results of the combined effect size by MD showed that there was still statistical significance in each subgroup, which further verified the reliability of the results (BI, MD = 9.02; 95% CI [2.39 to 15.64]; mBI, MD = 8.27; 95% CI [6.21 to 10.32]; other, MD = 11.20; 95% CI [-0.03 to 22.42]; unclear, MD = 8.23; 95% CI [1.79 to 14.67]) (Supplementary Figure S14).

#### 3.5.5 Volume of intracerebral hematoma (mL)

**FIGURE 8:**
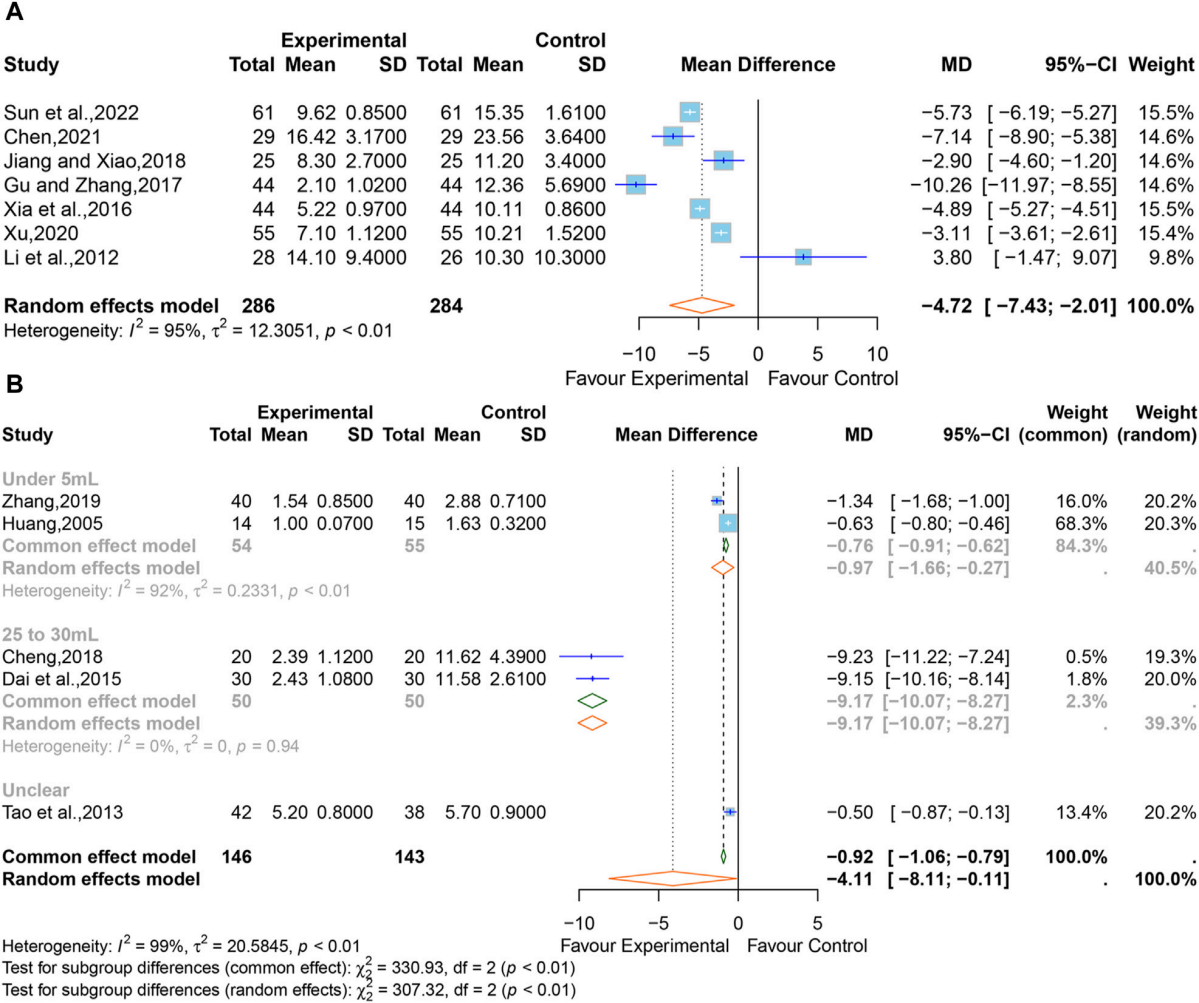
**(A)** Forest plot for the effect of Xingnaojing on the volume of intracerebral hematoma. **(B)** Forest plot for the effect of Xingnaojing on the volume of perihematomal edema.

A total of seven studies (Li et al., 2012; Xia et al., 2016; Gu and Zhang, 2017; Jiang and Xiao, 2018; Xu, 2020; Chen, 2021; Sun et al., 2022) comprising 570 participants reported the intracerebral hematoma volume. Considering that high heterogeneity (I^2^ = 92%, *p <* 0.01), a random-effects model was adopted, and the pooled data showed that XNJ reduced the hematoma volume (MD = −4.72; 95% CI [-7.43 to −2.01]; *p* = 0.0006] (Figure 8A). The subgroup analyses could not explain the source of the heterogeneity (Supplementary Table S4). Sensitivity analysis indicated that the combined effect size was stable (Supplementary Figure S7).

#### 3.5.6 Volume of perihematomal edema (mL)

Five studies (Huang, 2005; Tao et al., 2013; Dai et al., 2015; Cheng, 2018; Zhang, 2019) reported the volume of perihematomal edema. The pooled results indicated statistically significant differences (MD = −4.11; 95% CI [-8.11 to −0.11]; *p* = 0.0441) between the XNJ group and the control group and showed large heterogeneity (I^2^ = 99%, *p <* 0.01) (Figure 8B).

**FIGURE 9:**
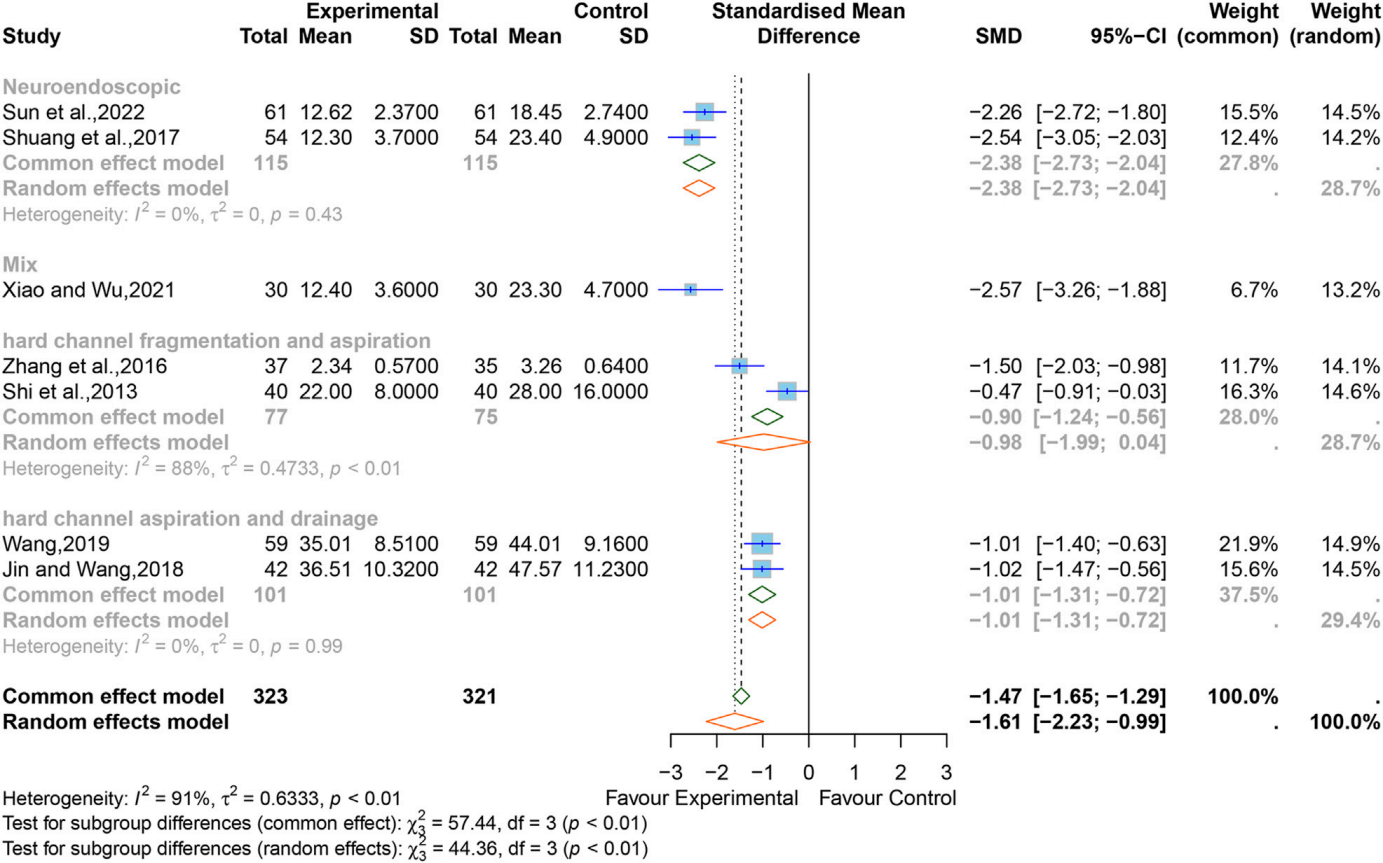
Forest plot for the effect of Xingnaojing on the levels of TNF-α.

The subgroup analyses based on “the volume of perihematomal edema before treatment” are shown in Figure 9. It can be seen that the heterogeneity in the group with 25–30 mL of perihematomal edema volume before treatment was significantly reduced (I^2^ = 0%, *p* = 0.94), whereas there was considerable heterogeneity in the group with less than 5 mL of perihematomal edema volume before treatment (I^2^ = 92%, *p <* 0.01), and one study did not specify the volume of perihematomal edema before treatment. The rest of the subgroup analyses are listed in Supplementary Table S5. Sensitivity analysis indicated that the combined effect size was stable (Supplementary Figure S8).

#### 3.5.7 Levels of TNF-α

Seven studies (Shi et al., 2013; Zhang et al., 2016; Shuang et al., 2017; Jiang and Xiao, 2018; Wang, 2019; Xiao and Wu, 2021; Sun et al., 2022) reported the levels of TNF-α. The SMD was used as a summary statistic due to the consistency of the units in these studies being unclear. The outcome showed statistically significant differences (SMD = −1.61, 95% CI [−2.23 to −0.99], *p <* 0.0001) (Figure 9) between the XNJ and the control group and indicated large heterogeneity (I^2^ = 91%, *p <* 0.01). Sensitivity analysis confirmed that the combined effect size was stable (Supplementary Figure S9).

Subgroup analyses based on different surgical techniques revealed that the heterogeneity was significantly reduced in the “neuroendoscopic hematoma evacuation surgery (NES)” group and the “hard channel puncture hematoma aspiration and drainage surgery (HCPADS)” group, while it was still high in the “hard channel puncture hematoma fragmentation and aspiration surgery (HCPFAS)” group (NES, I^2^ = 0%, *p =* 0.99; HCPADS, I^2^ = 0%, *p =* 0.43; HCPFAS, I^2^ = 88%, *p <* 0.01). The “mixed surgery” group (which included patients who underwent different surgical techniques) incorporated only one study. The rest of the subgroup analyses are listed in Supplementary Figure S6.

#### 3.5.8 Safety outcomes (adverse drug reactions, incidence of complications)

**FIGURE 10:**
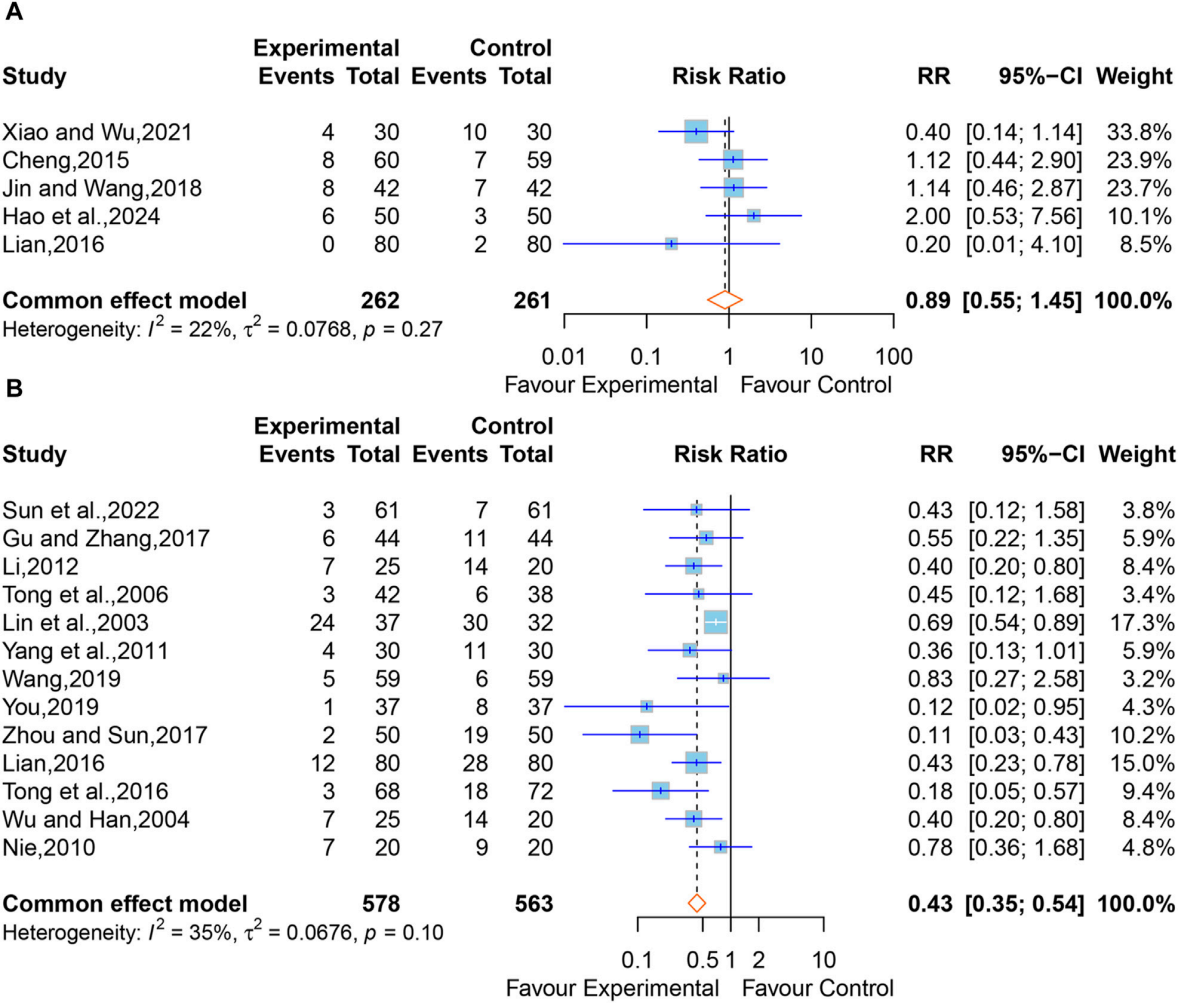
**(A)** Forest plot for the effect of Xingnaojing on adverse drug reactions. **(B)** Forest plot for the effect of Xingnaojing on incidence of complications.

In all, six studies reported adverse drug reactions following treatment. However, one study (Huang, 2005) merely stated that no adverse reactions or complications were observed in the XNJ group. We were unable to synthesize this study. The pooled results of the other five studies (Cheng, 2015; Lian, 2016; Jin and Wang, 2018; Xiao and Wu, 2021; Hao et al., 2024) clarified that there was no significant difference between the XNJ group and the control group (RR = 0.89; 95% CI [0.55 to 1.45]; *p* = 0.6521) (Figure 10A). Meanwhile, No heterogeneity was found (I^2^ = 22%, *p* = 0.27); thus, a fixed-effects model was adopted. Sensitivity analysis indicated that the overall effect was stable (Supplementary Figure S10).

Apart from one study (Huang, 2005) that only mentioned no complications in the XNJ group, 15 studies (Lin et al., 2003; Wu and Han, 2004; Tong et al., 2006; Nie, 2010; Yang et al., 2011; Li, 2012; Lian, 2016; Tong et al., 2016; Gu and Zhang, 2017; Zhou and Sun, 2017; Wang, 2019; You, 2019; Sun et al., 2022) reported the postoperative complications in two groups of post-operative patients of ICH. But two of them (Guo, 2015; Xu and Lv, 2015) reported data on the person-time of the outcome, we were unable to synthesize this study. Using the fixed-effect model (I^2^ = 35%, *p* = 0.10) for the meta-analysis of the remaining 13 studies, and the results demonstrated that XNJ significantly reduced the incidence of postoperative complications in post-operative patients of ICH (RR = 0.43; 95% CI [0.35 to 0.54]; *p* < 0.0001) (Figure 10B). Sensitivity analysis indicated that the overall effect was stable (Supplementary Figure S11). Specific adverse drug reactions and incidence of complications mentioned in the studies are listed in Supplementary Tables S7-S8.

### 3.6 Publication bias

The statistical test showed that no obvious publication bias was found in included trials regarding the all-cause mortality (Begg’s test, *p* = 0.8202; Egger’s test, *p* = 0.6864), the GCS score (Begg’s test, *p* = 0.5371; Egger’s test, *p* = 0.4034), the ADL (Begg’s test, *p* = 0.5371; Egger’s test, *p* = 0.2888), the hematoma volume (Begg’s test, *p* = 1.0000; Egger’s test, *p* = 0.8659), the volume of perihematomal edema (Begg’s test, *p* = 0.2207; Egger’s test, *p* = 0.0722), the TNF-α (Begg’s test, *p* = 0.1331; Egger’s test, *p* = 0.1485), and adverse drug reactions (Begg’s test, *p* = 0.8065; Egger’s test, *p* = 0.6051). However, a publication bias risk was present for the total efficiency rate (Begg’s test, *p* = 0.0030; Egger’s test, *p* = 0.0009), the neurological impairment (Begg’s test, *p* = 0.0351; Egger’s test, *p* = 0.0174), and the incidence of complications (Begg’s test, *p* = 0.1779; Egger’s test, *p* = 0.0069) (Table 2).

**TABLE 2:** Publication bias statistical test by Begg’s test and Egger’s test.

Outcomes	No. of studies	*p-*value	
		Begg’s test	Egger’s test
Total efficiency rate	25	0.0030	0.0009
All-cause mortality	18	0.8202	0.6864
Neurological impairment	31	0.0351	0.0174
Level of consciousness (GCS)	12	0.5371	0.4034
Activities of daily living	12	0.5371	0.2888
Volume of intracerebral hematoma	7	1.0000	0.8659
Volume of perihematomal edema	5	0.2207	0.0722
Levels of TNF-α	7	0.1331	0.1485
Adverse drug reactions	5	0.8065	0.6051
Incidence of complications	13	0.1779	0.0069

### 3.7 Results of TSA

**FIGURE 11:**
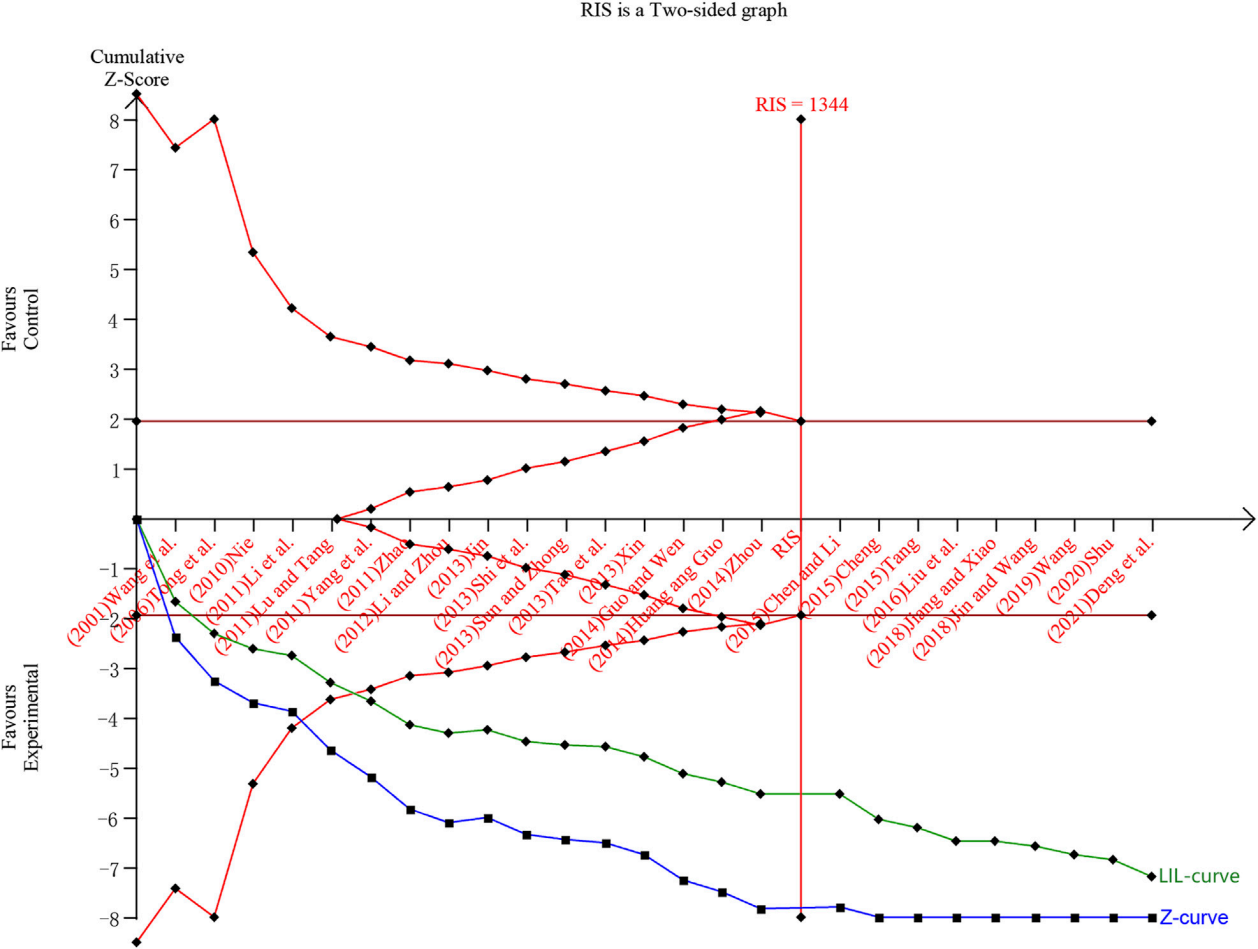
Trial sequential analysis and penalty statistics analysis of total efficiency rate. Note: The blue curve is the cumulative Z curve, the green curve (LIL-curve) is the penalized Z curve, the red horizontal line is the traditional threshold, the red curve is the TSA threshold, and the red vertical line is the RIS.

In this study, TSA analysis was performed on 25 studies that reported total effective rates which used “18% reduction in post-treatment neurological impairment scales scores” as the criterion, and the parameters were set according to the user manual for TSA (Thorlund et al., 2017), the type of boundary value was set as two-sided, type I error was defined as *α* = 0.05, statistical efficacy 1-*β* = 0.8. The results showed that the cumulative *Z*-value crossed the traditional boundary value (*Z* = 1.96) when included in study 1 (Wang et al., 2001), crossed the TSA boundary value when included in study 5 (Lu and Tang, 2011), and reached the RIS when included in study 17 (Chen and Li, 2015). The penalised *Z*-curve also crossed the traditional boundary value after the inclusion of study 2 (Tong et al., 2006), crossed the TSA boundary value when included study 6 (Yang et al., 2011) and reached the RIS when included in study 17 (Chen and Li, 2015) (Figure 11).

### 3.8 Quality of evidence

The certainty of the evidence of XNJ on all-cause mortality within 15 days, volume of perihematomal edema, levels of TNF-α, and adverse drug reactions was rated as “moderate”; that on total efficiency rate, ADL, intracerebral hematoma volume, and incidence of complications was “low”; and that on neurological impairment and the GCS score was “very low” (Table 3). We judged the quality of evidence as moderate to very low, mainly due to the high risk of bias, imprecision and the severe heterogeneity.

**TABLE 3:** GRADE evidence profiles.

Outcomes	Certainty assessment							Effect		Certainty	Importance
	No. of studies	Study design	Risk of bias	Inconsistency	Indirectness	Imprecision	Other considerations	Relative (95% CI)	Absolute (95% CI)		
Total efficiency rate	25	RCTs	Serious^a^	Not Serious	Not Serious	Not serious	Publication Bias Strongly Suspected^b^	RR 1.26 (1.21–1.32)	181 more per 1,000 (from 146 more to 222 more)	Low	Critical
All-cause mortality within 15 days	11	RCTs	Not Serious	Not Serious	Not Serious	Serious^c^	None	RR 0.45 (0.30-0.67)	81 fewer per 1,000 (from 104 fewer to 49 fewer)	Moderate	Important
Neurological impairment	31	RCTs	Serious^a^	Serious^d^	Not Serious	Not serious	Publication Bias Strongly Suspected^b^	-	SMD 1.44 SD fewer (1.78 fewer to 1.11 fewer)	Very low	Important
Level of consciousness (GCS)	12	RCTs	Serious^a^	Serious^d^	Not Serious	Serious^c^	None	-	MD 2.08 higher (1.22 higher to 2.93 higher)	Very low	Important
Activities of daily living	12	RCTs	Serious^a^	Serious^d^	Not Serious	Not serious	None	-	SMD 1.22 SD higher (0.78 higher to 1.66 higher)	Low	Important
Volume of intracerebral hematoma	7	RCTs	Not Serious	Serious^d^	Not Serious	Serious^c^	None	-	MD 4.72 lower (7.43 lower to 2.01 lower)	Low	Important
Volume of perihematomal edema	5	RCTs	Not Serious	Serious^d^	Not Serious	Not serious	None	-	MD 4.11 SD lower (8.11 lower to 0.11 lower)	Moderate	Important
Levels of TNF-α	7	RCTs	Not serious	Serious^d^	Not serious	Not serious	None	-	SMD 1.61 SD lower (2.23 lower to 0.99 lower)	Moderate	Important
Adverse drug reactions	5	RCTs	Not Serious	Not Serious	Not Serious	Serious^c^	None	RR 0.89 (0.55–1.45)	12 fewer per 1,000 (from 50 fewer to 50 more)	Moderate	Important
Incidence of complications	13	RCTs	Not Serious	Not Serious	Not Serious	Serious^c^	Publication Bias Strongly Suspected^c^	RR 0.43 (0.35–0.54)	183 fewer per 1,000 (from 209 fewer to 148 fewer)	Low	Important

^a^ Lack of blind method.

^b^ Significant publication bias was identified by Begg’s test and Egger’s test.

^c^ Some of the included studies had confidence intervals that crossed the line of equivalence.

^d^ There is significant heterogeneity among the included studies.

## 4 Discussion

### 4.1 Research significance

Intracerebral haemorrhage (ICH) is the most difficult to treat, the most disabling, and the deadliest type of stroke subtype (Tapia-Pérez et al., 2014; Wilkinson et al., 2018). The mechanisms of pathological damage after ICH primarily involve: the mass effect and mechanical rupture caused by the initial or ongoing bleeding and the expansion of the hematoma, which raise the overall pressure (intracranial pressure) and directly lead to primary brain injury; the physiological response to the hematoma (primarily edema and inflammation), the metabolic effects of thrombotic components, and secondary brain injury caused by toxic biochemicals (Wilkinson et al., 2018). One-third of ICH patients die within a month, and a large number of survivors are left with permanent disabilities (Steiner et al., 2011). Symptomatic treatment has been the primary treatment strategy for ICH to date (Tang et al., 2018). Surgery remains a vital measure for saving lives in emergency situations; however, the most common sites for ICH are deep brain structures, such as the basal ganglia and thalamus. Surgery requires passage through portions of brain tissue, which can lead to iatrogenic injury of healthy brain tissue. In addition, the presence of perihematomal edema after surgery may limit the therapeutic effect (Thompson et al., 2015; de Oliveira Manoel, 2020). Therefore, treatment targeting residual hematoma and cerebral edema post-operatively is a crucial aspect of care (Murthy et al., 2015; Chen et al., 2016).

Results from a systematic pharmacology study (Chen et al., 2018) suggest that XNJ might exert an anti-stroke effect by responding to oxidative stress, regulating blood pressure, calcium signaling pathways, and cell apoptosis among other biological processes and pathways, and Akt1, HIF1a, and ITGB2 may play key roles in the occurrence and regulation of stroke. 1,7-Diphenyl-3-acetoxy-6(E)-hepten, oxycurcumenol and beta-sitosterol may be essential compounds in XNJ and have been reported as effective ingredients for the treatment of stroke. The study also experimentally demonstrated that the oxycurcumenol has a protective effect on PC12 cells against oxidative stress-induced cellular damage. This mechanism does not involve cell cycle-dependent processes but may function through the regulation of autophagy, preliminarily unveiling the potential mechanisms by which XNJ treats stroke systematically. Our research further clarified the clinical efficacy and safety of XNJ in treating post-operative patients with ICH through an evidence-based evaluation, providing support for the clinical application of XNJ from an evidence-based perspective.

### 4.2 Summary of the main results

This meta-analysis included a total of 57 studies involving post-operative patients with ICH. It encompassed 2,445 cases that received a combination of XNJ with CWM treatment and 2,407 cases that received only CWM treatment. This study indicated that in comparison to other outcome indicators, authors of previous studies appeared to prefer to use the total efficiency rate rather than all-cause mortality as an endpoint indicator. And a few studies hold a dialectical perspective toward this phenomenon (Shi et al., 2023). This is due to the fact that the total efficiency rate, as a composite indicator, still lacks a universally accepted standardized evaluation method, and it is an insufficient strategy to evaluate a composite endpoint as if it were a sole primary endpoint (McCoy, 2018). However, we hold a conservative view on this because for patients, efficacy as a positive outcome may be more acceptable than mortality. And the “Clinical neurological impairment scoring standards for stroke patients” (NACCDC, 1996) formed at the fourth Chinese conference on cerebrovascular diseases in 1995 unified the criteria for assessing the “effectiveness” of stroke patient treatment, which was defined as a reduction in neurological impairment score of ≥18% after treatment. The results of this study showed that most of the previous studies used the aforementioned assessment method to evaluate the total efficiency rate. However, many studies also used different efficacy assessment criteria, and we found through subgroup analyses that there was no significant difference between results using different criteria, and there was low heterogeneity in the overall effect of the meta-analysis.

As for the outcome indicators of neurological impairment and ADL, there were similar issues, especially regarding the assessment of ADL. Some studies only mentioned the use of ADL scales but did not specify the names and criteria of the scales used. In fact, there were many scales commonly used to assess ADL, such as the BI and mBI, etc. We merged the effect sizes of all the studies included in the outcome indicators of neurological impairment and ADL through SMD and compared them with results obtained by merging effect sizes using MD. The results showed that XNJ could significantly reduce neurological impairment and improve ADL after treatment. In contrast, regarding the consciousness state, all studies used GCS for evaluation, and results showed that XNJ significantly improved GCS scores after treatment, but there was also obvious heterogeneity between studies.

Compared with the above subjective outcome indicators, this study also included some objective outcome indicators, and the results showed that XNJ significantly reduced all-cause mortality, hematoma volume, perihematomal edema, and the inflammatory marker TNF-α after treatment. However, subgroup analyses indicated that XNJ had a significant effect on reducing all-cause mortality at 2 weeks or 15 days after starting treatment, but could not reduce all-cause mortality at 4 weeks or 1 month, and even longer time points by pooling a few corresponding data, although there is still a lack of sufficient research to prove its therapeutic effect on 6-month mortality. Despite this, the outcome is still encouraging, as so far, no intervention has demonstrated improved outcomes. Additionally, we conducted specific analyses on safety indicators. Although some studies mentioned adverse reactions, we found that they include two situations: drug adverse reactions and postoperative complications. Our specific analysis showed that XNJ could significantly reduce the incidence of postoperative complications after the surgery of ICH without increasing drug adverse reactions.

Due to the influence of many confounding factors such as surgical methods, geographical regions, age, and methods of outcome evaluation, significant heterogeneity existed among studies included for outcome indicators other than the overall efficacy rate, all-cause mortality, and safety metrics. Despite conducting subgroup and sensitivity analyses, we still cannot completely rule out the impact of confounding factors on the results. Subgroup analyses revealed that the heterogeneity was significantly reduced in the ESS group for the outcome of neurological impairment and in the mBI group for the outcome of ADL. The Subgroup analyses regarding GCS found lower heterogeneity among studies conducted in major affiliated hospitals of the university but yielded negative results, which may be due to the fact that the included patients were those with complex or more severe conditions due to the higher hospital level, thus limiting the treatment effect. The subgroup analyses on perihematomal edema volume showed higher heterogeneity in the group with postoperative edema volume of less than 5 mL, which may be related to the larger measurement errors associated with lower edema volumes. The subgroup analyses targeting TNF-α found that the NES group and the HCPADS group had lower heterogeneity and that the NES group achieved better therapeutic effects. The sources of heterogeneity in the remaining subgroup analyses could not be well explained. Moreover, the results of TSA showed that the cumulative Z-value and the penalised Z-curve crossed both the traditional boundaries and the TSA boundaries, reached the RIS, led to a positive conclusion and excluding the possibility of false positives. Unfortunately, due to the high level of heterogeneity and the absence of blinding in subjective outcome indicators, the level of evidence for the study results is generally low, and our findings according to the current studies should be considered carefully in the clinic.

### 4.3 Strengths and limitations

Compared to the previous network meta-analysis concerning post-operative patients with ICH (Ren et al., 2022), this meta-analysis included the latest RCTs. Past network meta-analyses only focused on the total efficiency rate, NIHSS, and intracerebral hematoma volume. However, we attempted to investigate whether XNJ could reduce all-cause mortality, perihematomal edema volume, TNF-α, and improve the ADL, which are more objective and important for post-operative patients with ICH. We comprehensively collected and assessed existing research data for each outcome indicator, despite that they adopted different evaluation methods for the same outcome indicator. Moreover, we conducted subgroup analyses for different evaluation methods and displayed the results for the convenience of clinical specialists and other researchers’ access. In addition, this study performed more comprehensive subgroup analyses for outcome indicators with high heterogeneity, to interpret sources of the heterogeneity and the efficacy results, and explored the stability of the results through sensitivity analyses, etc. Previously, there have been no conventional meta-analysis studies published that specifically involve the use of TCM injections in post-operative patients with ICH.

This study also has certain limitations: the included 57 studies were mostly single-center and small-sample research; some studies only mentioned random allocation without specifying the exact methods; the surgical methods, efficacy evaluation criteria, and outcome indicators varied among the studies; the studies reported only short-term mortality rates, lacking long-term prognosis follow-up, etc. At the same time, the presence of significant publication bias might also affect the reliability of the results.

In view of the above limitations, future research should strengthen the integrity of experimental designs, pay special attention to the accurate application of random methods, allocation concealment, and blinding, clearly define long-term efficacy and safety, and to the extent possible choose widely recognized, unified outcome indicators (Liu et al., 2018), etc. Considering these limitations, the results of this study still await further high-quality RCT research to provide more reliable evidence-based support.

## 5 Conclusion

In conclusion, the present meta-analysis and systematic review of 57 RCTs indicates that the administration of XNJ for post-operative patients with ICH is associated with favorable short-term outcomes (within 1 moth). And it can improve total efficiency rate, level of consciousness, and activities of daily living; alleviate neurological impairment; reduce all-cause mortality, volume of cerebral hematoma, volume of perihematomal edema, levels of TNF- α, incidence of complications, and has good tolerability. However, The current evidence base is insufficient and requires substantiation from further high-quality studies. Methodological shortcomings and a substantial risk of bias have curtailed the positive effects, undermining confidence in the synthesis of evidence. Given the preliminary nature of the evidence and that XNJ has enormous potential as a therapeutic agent for ICH, it is imperative to conduct more stringent RCTs to validate the efficacy of XNJ in post-operative patients with ICH.

## Data Availability

The original contributions presented in the study are included in the article/Supplementary Material, further inquiries can be directed to the corresponding authors.
