# Supplementary material for: Efficacy and safety of Xingnaojing injection for post-operative patients of intracerebral haemorrhage: a meta-analysis and systematic review
Source: Front Pharmacol. 2024 Jun 5;15:1411026. doi: 10.3389/fphar.2024.1411026 (PMC11185956; doi:10.3389/fphar.2024.1411026)
Supplement: Supplementary file 1 [file DataSheet1.pdf]

Supplementary Material

Table of contents

1 Supplementary Tables 3

Supplementary Table S1. Subgroup analyses of Xingnaojing on the neurological impairment 3

Supplementary Table S2. Subgroup analyses of Xingnaojing on the Glasgow coma scale 4

Supplementary Table S3. Subgroup analyses of Xingnaojing on the activities of daily living 6

Supplementary Table S4. Subgroup analyses of Xingnaojing on the intracerebral hematoma volume 7

Supplementary Table S5. Subgroup analyses of Xingnaojing on the volume of perihematomal edema 8

Supplementary Table S6. Subgroup analyses of Xingnaojing on TNF-α........ 9

Supplementary Table S7. Adverse drug reactions 10

Supplementary Table S8. Incidence of complications 11

2 Supplementary Figures 12

Supplementary Figure S1. The risk of bias summary for each study 12

Supplementary Figure S2. Forest plot of sensitivity analysis of Xingnaojing on total efficiency rate (18% reduction in post-treatment neurological impairment scales scores) 13

Supplementary Figure S3. Forest plot of sensitivity analysis of Xingnaojing on the mortality rate. 13

Supplementary Figure S4. Forest plot of sensitivity analysis of Xingnaojing on the neurological impairment 13

Supplementary Figure S5. Forest plot of sensitivity analysis of Xingnaojing on the Glasgow coma scale........ 14

Supplementary Figure S6. Forest plot of sensitivity analysis of Xingnaojing on the activities of daily living 14

Supplementary Figure S7. Forest plot of sensitivity analysis of Xingnaojing on the intracerebral hematoma volume........ 14

Supplementary Figure S8. Forest plot of sensitivity analysis of Xingnaojing on the volume of perihematomal edema........ 15

Supplementary Figure S9. Forest plot of sensitivity analysis of Xingnaojing on TNF-a 15

Supplementary Figure S10. Forest plot of sensitivity analysis of Xingnaojing on adverse drug reactions 15

Supplementary Figure S11. Forest plot of sensitivity analysis of Xingnaojing on incidence of complications 15

Supplementary Figure 12. Forest plot of Xingnaojing on total efficiency rate assessed by different criteria 16

Supplementary Figure S13. Forest plot of Xingnaojing on neurological impairment assessed by different scales (MD) 17

Supplementary Figure S14. Forest plot of Xingnaojing on the Activities of Daily Living assessed by different scales (MD) 17

3 Supplementary File 19

Supplementary File S1. Search strategy 19

Supplementary File S2. The PRISMA checklist of this meta-analysis 23

1 Supplementary Tables

Supplementary Table S1. Subgroup analyses of Xingnaojing on the neurological impairment.

Grouping criteria	subgroup	No.	Effect model	*SMD*(95%*Cl*)	Heterogeneity test	
					P-value	I^2(%)
Disease	HICH	11	Random	-1.65(-2.54, -0.76)	<0.01	95
	ICH	20	Random	-1.33(-1.57, -1.09)	<0.01	90
Age (year)	17 to 82	2	Random	-1.58(-2.27, -0.44)	<0.01	86
	30 to 81	4	Random	-2.47(-4.61, -0.34)	<0.01	97
	40 to 82	11	Random	-1.10(-1.42, -0.79)	<0.01	82
	50 to 82	8	Random	-1.61(-2.21, -1.01)	<0.01	90
	Over 60	2	Fixed	-0.92(-1.23, -0.61)	0.24	26
	Unclear	4	Random	-1.35(-1.96, -0.74)	<0.01	87
Average intracerebral hematoma volume (ml)	20 to 30	4	Random	-1.46(-2.04, -0.88)	<0.01	82
	30 to 40	4	Random	-1.19(-1.77, -0.62)	<0.01	80
	40 to 50	2	Random	-1.78(-2.43, -1.13)	0.06	71
	50 to 60	7	Random	-1.46(-2.11, -0.80)	<0.01	92
	60 to 70	1	Random	-1.46(-1.91, -1.02)	-	-
	Unclear	13	Random	-1.47(-2.20, -0.74)	<0.01	93
Course of disease	within 24h	8	Random	-1.21(-1.56, -0.87)	<0.01	76
	within 36h	2	Random	-2.07(-2.73, -1.40)	0.12	58
	within 48h	4	Random	-1.03(-1.79, -0.26)	<0.01	88
	within 72h	4	Random	-2.62(-4.78, -0.46)	<0.01	98
	Unclear	13	Random	-1.28(-1.60, -0.96)	<0.01	82
Dosage (ml)	10	1	Random	-1.50(-1.94, -1.07)	-	-
	4	1	Random	-0.86(-1.63, -0.10)	-	-
	10 to 20	1	Random	-0.67(-1.19, -0.15)	-	-
	20	15	Random	-1.68(-2.32, -1.05)	<0.01	93
	20 to 30	2	Fixed	-0.95(-1.25, -0.64)	0.94	0
	30	7	Random	-1.37(-2.01, -0.73)	<0.01	92
	40	4	Random	-1.28(-1.66, -0.89)	0.03	65
Duration of treatment	1w	3	Random	-1.10(-2.10, -0.09)	<0.01	92
	2w	19	Random	-1.24(-1.50, -0.98)	<0.01	81
	3w	1	Random	-1.67(-2.03, -1.31)	-	-
	4w or 1m	6	Random	-2.21(-3.63, -0.79)	<0.01	96
	6w	1	Random	-0.65(-1.05, -0.25)	-	-
	Unclear	1	Random	-2.59(-3.13, -2.05)	-	-
Observation	1w	3	Random	-1.10(-2.10, -0.09)	<0.01	92
	2w	15	Random	-1.23(-1.51, -0.95)	<0.01	80
	4w or 1m	10	Random	-1.85(-2.75, -0.94)	<0.01	94
	3w	1	Random	-1.67(-2.03, -1.31)	-	-

Supplementary Table S1. (*Continued*) Subgroup analyses of Xingnaojing on the neurological impairments.

Grouping criteria	subgroup	No.	Effect model	*SMD*(95%*Cl*)	Heterogeneity test	
					P-value	I^2(%)
Observation	6w	1	Random	-0.65(-1.05, -0.25)	-	-
	Unclear	1	Random	-2.59(-3.13, -2.05)	-	-
Surgery	SCPADS	6	Random	-0.93(-1.41, -0.45)	<0.01	82
	HCPADS	8	Random	-1.85(-2.22, -1.48)	<0.01	82
	HCPFAS	4	Random	-0.77(-1.19, -0.36)	0.03	66
	PADS	1	Random	-2.45(-2.92, -1.97)	-	-
	NES	3	Random	-1.48(-1.93, -1.03)	0.03	72
	Mix	2	Random	-3.23(-8.26, 1.80)	<0.01	99
	Unclear	7	Fixed	-1.19(-1.37, -1.01)	0.28	20
Area	Municipal hospital	16	Random	-1.90(-2.46, -1.34)	<0.01	92
	Military hospital	2	Random	-0.73(-1.19, -0.27)	0.13	56
	County hospital	7	Fixed	-0.96(-1.13, -0.78)	0.30	17
	Affiliated hospital of university	4	Random	-1.06(-1.71, -0.40)	<0.01	85
	Others	2	Random	-1.07(-1.85, -0.28)	0.01	84

Supplementary Table S2. Subgroup analyses of Xingnaojing on the Glasgow coma scale.

Grouping criteria	subgroup	No.	Effect model	*MD*(95%*Cl*)	Heterogeneity test	
					P-value	I^2(%)
Disease	HICH	4	Random	0.97(-0.25, 2.20)	<0.01	75
	ICH	8	Random	2.63(1.72, 3.53)	<0.01	86
Age (year)	40 to 82	5	Random	1.94(-0.11, 3.98)	<0.01	93
	50 to 82	3	Fixed	2.18(1.85, 2.50)	0.37	0
	Over 60	2	Fixed	2.56(1.80, 3.32)	0.97	0
	Unclear	2	Random	1.97(-0.57, 4.52)	<0.01	92
Average intracerebral hematoma volume (ml)	20 to 30	1	-	0.73(0.04, 1.42)	-	-
	30 to 40	2	Random	2.60(-0.25, 5.45)	<0.01	89
	40 to 50	1	-	-0.45(-1.80, 0.90)	-	-
	50 to 60	4	Random	2.08(0.30, 3.85)	<0.01	89
	Unclear	4	Fixed	2.72(2.10, 3.34)	0.76	0
Course	within 24h	3	Random	1.06(-0.73, 2.86)	<0.01	93
	within 48h	1	-	0.70(-2.53, 1.13)	-	-
	within 72h	3	Random	2.90(1.55, 4.24)	<0.01	87
	Unclear	5	Random	2.55(1.46, 3.65)	<0.01	93
Dosage (ml)	10 to 20	1	-	2.57(1.55, 3.59)	-	-
	20	4	Random	2.35(0.38, 4.33)	<0.01	87
	20 to 30	1	-	1.00(-0.84, 2.84)	-	-
	30	5	Random	2.17(0.82, 3.52)	<0.01	88

Supplementary Table S2. (*Continued*) Subgroup analyses of Xingnaojing on the Glasgow coma scale.

Grouping criteria	subgroup	No.	Effect model	*MD*(95%*Cl*)	Heterogeneity test	
					P-value	I^2(%)
Dosage (ml)	40	1	-	0.73(0.04, 1.42)	-	-
Duration of treatment/observation	1w	2	Random	1.00(-2.17, 4.17)	<0.01	88
	2w	7	Random	1.95(0.81, 3.09)	<0.01	92
	3w	1	-	4.10(3.21, 4.99)	-	-
	6w	1	-	2.40(0.51, 4.29)	-	-
	Unclear	1	-	2.31(1.85, 2.77)	-	-
Surgery	SCPADS	2	Random	1.32(-0.24, 2.89)	0.10	62
	HCPADS	3	Random	3.18(2.07, 4.30)	<0.01	85
	HCPFAS	2	Fixed	0.15(-1.15, 1.44)	0.20	40
	PADS	1	-	2.11(1.63, 2.59)	-	-
	NES	1	-	2.54(1.40, 3.68)	-	-
	Craniotomy	1	-	-0.45(-1.80, 0.90)	-	-
	Mix	1	-	2.57(1.55, 3.59)	-	-
	Unclear	1	-	3.92(3.38, 4.46)	-	-
Before	GCS≤8	5	Random	2.74(1.16, 4.33)	<0.01	90
	8<GCS≤11	7	Random	1.62(0.85, 2.40)	<0.01	76
Area	Municipal hospital	7	Random	2.72(2.15, 3.30)	<0.01	65
	County hospital	2	Random	2.60(-0.25, 5.45)	<0.01	89
	Affiliated hospital of university	3	Fixed	0.09(-0.88, 1.07)	0.15	47

Supplementary Table S3. Subgroup analyses of Xingnaojing on the activities of daily living.

Grouping criteria	subgroup	No.	Effect model	*MD*(95%*Cl*)	Heterogeneity test	
					P-value	I^2(%)
Disease	HICH	10	Random	10.32(6.88, 13.77)	<0.01	90
	ICH	2	Random	3.53(-1.24, 8.30)	<0.01	89
Age (year)	17 to 82	1	-	0.53(-0.11, 1.16)	-	-
	30 to 81	1	-	1.89(1.39, 2.40)	-	-
	40 to 82	3	Fixed	1.32(1.02, 1.62)	0.28	22
	50 to 82	6	Random	1.21(0.33, 2.09)	<0.01	91
	Unclear	1	-	1.10(0.48, 1.73)	-	-
Average intracerebral hematoma volume (ml)	20 to 30	2	Fixed	0.82(0.37, 1.26)	0.20	38
	40 to 50	3	Random	1.10(0.41, 1.80)	<0.01	81
	50 to 60	1	-	1.89(1.39, 2.40)	-	-
	60 to 70	1	-	1.63(1.17, 2.08)	-	-
	Unclear	5	Random	1.27(0.27, 2.27)	<0.01	91
Course	within 24h	3	Random	0.74(-0.02, 1.51)	<0.01	86
	within 36h	1	-	3.25(2.37, 4.13)	-	-
	within 48h	1	-	1.69(1.18, 2.20)	-	-
	Unclear	7	Random	1.12(0.72, 1.53)	<0.01	79
Dosage (ml)	10 to 20	1	-	0.43(-0.13, 0.99)	-	-
	20	4	Random	-1.62(0.53, 2.71)	<0.01	89
	30	2	Random	0.96(-0.05, 1.96)	<0.01	90
	40	3	Fixed	1.60(1.30, 1.90)	0.15	47
	20 to 40	1	-	1.18(0.68, 1.67)	-	-
	Based on weight	1	-	0.21(-0.27, 0.68)	-	-
Duration of treatment	2w	10	Random	1.18(0.65, 1.71)	<0.01	87
	3w	1	-	1.18(0.68, 1.67)	-	-
	4w or 1m	1	-	1.69(1.18, 2.20)	-	-
Observation	2w	6	Random	0.97(0.41, 1.53)	<0.01	86
	3w	1	-	1.18(0.68, 1.67)	-	-
	4w or 1m	5	Random	1.56(0.70, 2.41)	<0.01	89
Surgery	SCPADS	5	Random	0.80(0.37, 1.23)	<0.01	71
	HCPADS	2	Random	2.43(0.90, 3.96)	<0.01	89
	HCPFAS	1	-	1.89(1.39, 2.40)	-	-
	Mix	1	-	0.21(-0.27, 0.68)	-	-
	Unclear	3	Fixed	1.32(1.02, 1.62)	0.28	22
Area	Municipal hospital	4	Random	1.68(0.58, 2.78)	<0.01	89
	County hospital	3	Random	1.19(0.47, 1.91)	<0.01	83
	Affiliated hospital of university	2	Random	0.63(-0.25, 1.51)	0.03	80
	Others	2	Random	0.81(0.11, 1.51)	0.02	81

Supplementary Table S4. Subgroup analyses of Xingnaojing on the intracerebral hematoma volume.

Grouping criteria	subgroup	No.	Effect model	*MD*(95%*Cl*)	Heterogeneity test	
					P-value	I^2(%)
Disease	HICH	5	Random	-5.34(-7.85, -2.82)	<0.01	96
	ICH	2	Random	-1.96(-12.67, 8.74)	<0.01	93
Age (year)	17 to 82	1	-	-10.26(-11.97, -8.55)	-	-
	30 to 81	1	-	-4.89(-5.27, -4.51)	-	-
	40 to 82	2	Random	-1.96(-12.67, 8.74)	<0.01	93
	50 to 82	1	-	-2.90(-4.60, -1.20)	-	-
	Over 60	1	-	-5.73(-6.19, -5.27)	-	-
	Unclear	1	-	-3.11(-3.61, -2.61)	-	-
Average intracerebral hematoma volume (ml)	20 to 30	1	-	-10.26(-11.97, -8.55)	-	-
	30 to 40	2	Random	-5.03(-8.98, -1.09)	<0.01	95
	40 to 50	1	-	-2.90(-4.60, -1.20)	-	-
	50 to 60	2	Random	-0.96(-9.44, 7.52)	<0.01	90
	Unclear	1	-	-5.73(-6.19, -5.27)	-	-
Course	within 24h	2	Random	-4.42(-6.99, -1.85)	<0.01	98
	within 48h	1	-	3.80(-1.47, 9.07)	-	-
	Unclear	4	Random	-6.27(-9.32, -3.21)	<0.01	94
Dosage (ml)	10 to 20	1	-	-2.90(-4.60, -1.20)	-	-
	20	4	Random	-4.53, -10.09,1.03	<0.01	96
	30	1	-	-5.73(-6.19, -5.27)	-	-
	40	1	-	-4.89(-5.27, -4.51)	-	-
Duration of treatment/observation	1w	3	Random	-2.27(-7.25, 2.71)	<0.01	97
	2w	4	Random	-6.27(-9.32, -3.21)	<0.01	94
Surgery	SCPADS	2	Random	-6.58(-13.79, 0.63)	<0.01	97
	HCPADS	1	-	-3.11(-3.61, -2.61)	-	-
	HCPFAS	2	Random	-0.96(-9.44, 7.52)	<0.01	90
	NES	1	-	-5.73(-6.19, -5.27)	-	-
	Unclear	1	-	-7.14(-8.90, -5.38)	-	-
Area	Municipal hospital	4	Random	-5.91(-8.80, -3.03)	<0.01	97
	County hospital	2	Random	-5.01(-9.17, -0.86)	<0.01	91
	Affiliated hospital of university	1	-	3.80(-1.47, 9.07)	-	-

Supplementary Table S5. Subgroup analyses of Xingnaojing on the volume of perihematomal edema.

Grouping criteria	subgroup	No.	Effect model	*MD*(95%*Cl*)	Heterogeneity test	
					P-value	I^2(%)
Disease	HICH	4	Random	-4.82(-9.70, 0.06)	<0.01	99
	ICH	1	-	-1.34(-1.68, -1.00)	-	-
Age (year)	17 to 82	2	Fixed	-9.17(-10.07, -8.27)	0.94	0
	30 to 81	1	-	-1.34(-1.68, -1.00)	-	-
	40 to 82	1	-	-0.50(-0.87, -0.13)	-	-
	50 to 82	1	-	-0.63(-0.80, -0.46)	-	-
Average intracerebral hematoma volume (ml)	20 to 30	2	Random	-5.22(-12.95, 2.51)	<0.01	98
	30 to 40	1	-	-0.63(-0.80, -0.46)	-	-
	50 to 60	1	-	-0.50(-0.87, -0.13)	-	-
	Unclear	1	-	-9.15(-10.16, -8.14)	-	-
Course	within 24h	2	Random	-4.87(-13.30, 3.56)	<0.01	99
	Unclear	3	Random	-3.64(-9.02, 1.73)	<0.01	99
Dosage (ml)	10	1	-	-0.63(-0.80, -0.46)	-	-
	10 to 20	1	-	-1.34(-1.68, -1.00)	-	-
	20	2	Fixed	-9.17(-10.07, -8.27)	0.94	0
	30	1	-	-0.50(-0.87, -0.13)	-	-
Duration of treatment/observation	2w	5	Random	-4.11(-8.11, -0.11)	<0.01	99
Surgery	SCPADS	2	Random	-5.23(-12.88, 2.42)	<0.01	100
	HCPADS	2	Random	-4.81(-13.36, 3.75)	<0.01	99
	Unclear	1	-	-0.63(-0.80, -0.46)	-	-
Before (ml)	Under 5	2	Random	-0.97(-1.66, -0.27)	<0.01	92
	25 to 30mL	2	Fixed	-9.17(-10.07, -8.27)	0.94	0
	Unclear	1	-	-0.50(-0.87, -0.13)	-	-
Area	Municipal hospital	3	Random	-6.52(-11.70, -1.34)	<0.01	99
	County hospital	1	-	-0.63(-0.80, -0.46)	-	-
	Military hospital	1	-	-0.50(-0.87, -0.13)	-	-

Supplementary Table S6. Subgroup analyses of Xingnaojing on TNF-α.

Grouping criteria	subgroup	No.	Effect model	*MD*(95%*Cl*)	Heterogeneity test	
					P-value	I^2(%)
Disease	HICH	6	Random	-1.46(-2.10, -0.82)	<0.01	91
	ICH	1	-	-2.57(-3.26, -1.88)	-	-
Age (year)	17 to 82	1	-	-0.47(-0.91, -0.03)	-	-
	40 to 82	1	-	-2.54(-3.05, -2.03)	-	-
	50 to 82	1	-	-1.50(-2.03, -0.98)	-	-
	Over 60	2	Fixed	-2.35(-2.74, -1.97)	0.47	0
	Unclear	2	Fixed	-1.01(-1.31, -0.72)	0.99	0
Average intracerebral hematoma volume (ml)	20 to 30	2	Fixed	-1.01(-1.31, -0.72)	0.99	0
	Unclear	5	Random	-1.86(-2.65, -1.07)	<0.01	92
Course	within 24h	3	Random	-1.60(-2.32, -0.87)	<0.01	86
	within 36h	2	Random	-1.50(-3.56, 0.56)	<0.01	96
	within 48h	1	-	-2.54(-3.05, -2.03)	-	-
	Unclear	1	-	-1.01(-1.40, -0.63)	-	-
Dosage (ml)	10 to 20	1	-	-2.57(-3.26, -1.88)	-	-
	20	4		-1.51(-2.21, -0.81)	<0.01	88
	30	1	-	-2.26(-2.72, -1.80)	-	-
	40	1	-	-0.47(-0.91, -0.03)	-	-
Duration of treatment/observation	1w	1	-	-2.26(-2.72, -1.80)	-	-
	2w	3	Random	-1.49(-2.67, -0.31)	<0.01	92
	1m	3	Random	-1.51(-2.50, -0.53)	<0.01	92
Surgery	HCPADS	2	Fixed	-1.01(-1.31, -0.72)	0.99	0
	HCPFAS	2	Random	-0.98(-1.99, 0.04)	<0.01	88
	NES	2	Fixed	-2.38(-2.73, -2.04)	0.43	0
	Mix	1	-	-2.57(-3.26, -1.88)	-	-
Area	Municipal hospital	4	Random	-1.95(-2.94, -0.95)	<0.01	94
	County hospital	1	-	-1.01(-1.40, -0.63)	-	-
	Military hospital	1	-	-1.50(-2.03, -0.98)	-	-
	Affiliated hospital of university	1	-	-1.02(-1.47, -0.56)	-	-

Supplementary Table S7. Adverse drug reactions.

Studies	No. of patients	
	Experimental	Control
Hao et al., 2024	Skin itching 4, tachycardia 1, chest tightness 1	Skin itching 3, tachycardia 0, chest tightness 0
Xiao and Wu, 2021	Headache 2, nausea 1, rash 1	Headache 4, nausea 3, rash 3
Jin and Wang, 2018	Diarrhea 2, headache 2, abnormal body temperature 3, abdominal pain 1	Diarrhea 2, headache 1, abnormal body temperature 2, abdominal pain 2
Lian, 2016	0	2
Cheng, 2015	Nausea, vomiting 3, skin itching 2, dizziness 2, tachycardia 1	Nausea, vomiting 4, arrhythmia 1, irritability 2 (symptoms disappear after slowing down the infusion rate or stopping the medication. Nausea and vomiting cannot be ruled out as caused by elevated intracranial pressure, and no serious adverse reactions were found in both groups)
Huang, 2005	no adverse drug reactions	-

Supplementary Table S8. Incidence of complications.

Studies	No. of patients	
	Experimental	Control
Sun et aL., 2022	Lower limb deep vein thrombosis 1, pressure ulcer 1, pain 1, other 0	Lower limb deep vein thrombosis 2, pressure ulcer 2, pain 2, other 1
Wang, 2019	Fever 2, hydrocephalus 2, chills 1	Fever 1, hydrocephalus 3, chills 2
You, 2019	Intracranial infection 1, functional impairment 0, cognitive impairment 0	Intracranial infection 2, functional impairment 4, cognitive impairment 2
Gu and Zhang, 2017	Fever 3, upper gastrointestinal bleeding 3	Fever 2, upper gastrointestinal bleeding 9
Zhou and Sun, 2017	Hydrocephalus 1, fever 1	Hydrocephalus 6, fever 10, intracranial infection 3
Lian, 2016	Fever 7, muscle weakness 2, pulmonary infection 3	Fever 17, muscle weakness 5, pulmonary infection 6
Tong et aL., 2016	Hydrocephalus 1, fever 2	Hydrocephalus 5, intracranial infection 4, fever 9
Yang et aL., 2011	Pulmonary infection 1, pulmonary infection with stress ulcer 2, renal insufficiency 1	Pulmonary infection 5, stress ulcer 3, pulmonary infection with stress ulcer 2, renal insufficiency 1
Tong et aL., 2006	The rebleeding after ICH surgery 3	The rebleeding after ICH surgery 6
Wu and Han, 2004	Pulmonary infection 2, urinary tract infection 2, upper gastrointestinal bleeding 1, high fever (T > 39°C) 2	Pulmonary infection 4, urinary tract infection 3, upper gastrointestinal bleeding 2, high fever (T > 39°C) 5
Lin et aL., 2003	Pulmonary infection, increased respiratory secretions requiring tracheostomy 15, upper gastrointestinal bleeding 9	Pulmonary infection, increased respiratory secretions requiring tracheostomy 21, upper gastrointestinal bleeding 9
Xu ang Lv, 2015	Hydrocephalus 9, pulmonary infection 20, hemiplegia aphasia 46, gastrointestinal bleeding 13, fever 14 (person-time)	Hydrocephalus 3, pulmonary infection 8, hemiplegia aphasia 39, gastrointestinal bleeding 4, fever 13 (person-time)
Guo, 2015	Pulmonary infection 8, hemiplegia and aphasia 28, gastrointestinal bleeding 4, fever 12 (person-time)	Pulmonary infection 15, hemiplegia and aphasia 36, gastrointestinal bleeding 10, fever 33 (person-time)
Li, 2012	Incidence of complications 7 person-time	Incidence of complications 14 person-time
Nie, 2010	Incidence of complications 7 person-time	Incidence of complications 9 person-time
Huang, 2005	no hematoma enlargement, no rebleeding, no worsening of the condition	-

2 Supplementary Figures

Supplementary Figure S1. The risk of bias summary for each study.

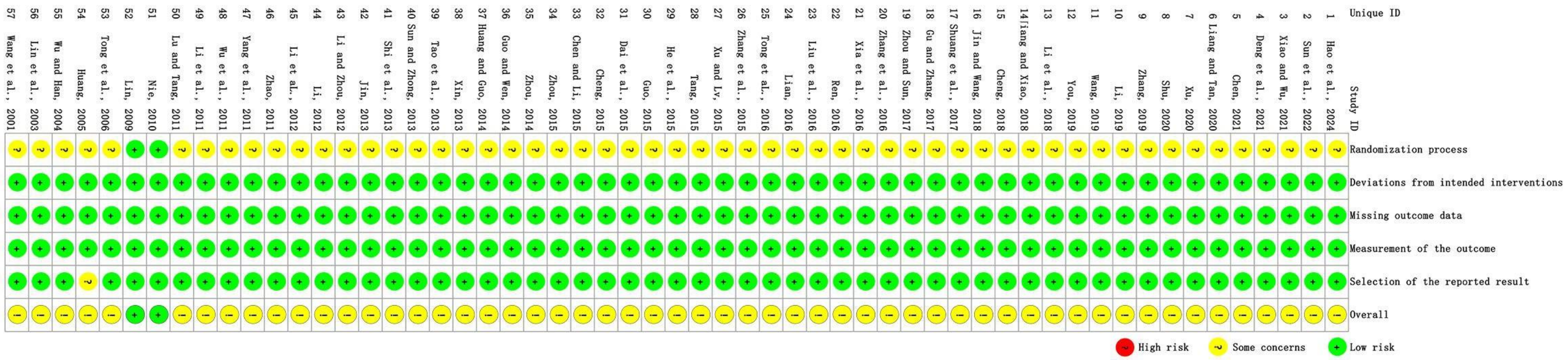

Supplementary Figure S2. Forest plot of sensitivity analysis of Xingnaojing on total efficiency rate (18% reduction in post-treatment neurological impairment scales scores).

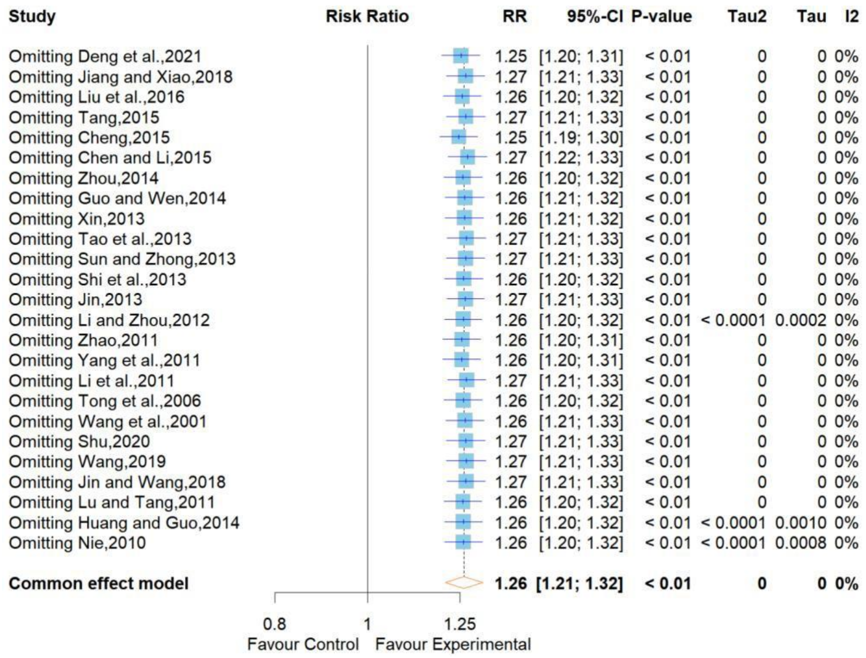

Supplementary Figure S3. Forest plot of sensitivity analysis of Xingnaojing on the mortality rate.

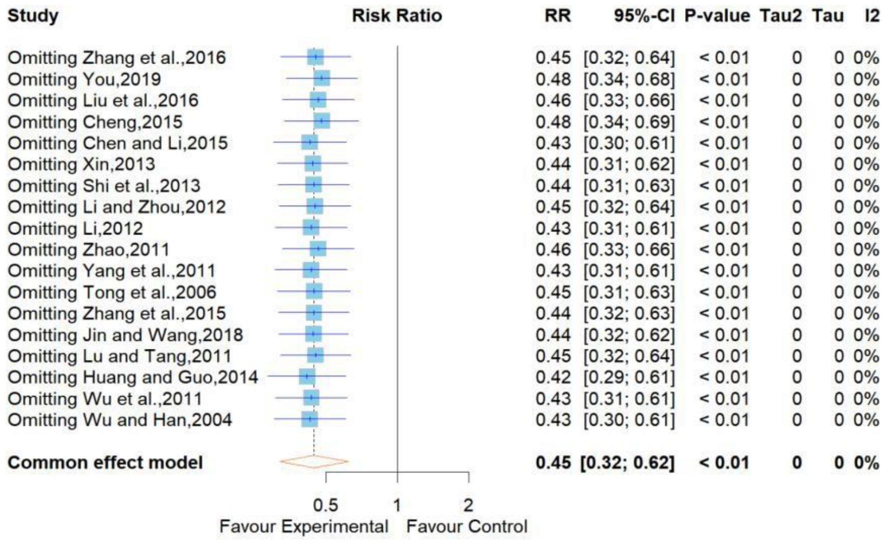

Supplementary Figure S4. Forest plot of sensitivity analysis of Xingnaojing on the neurological impairment.

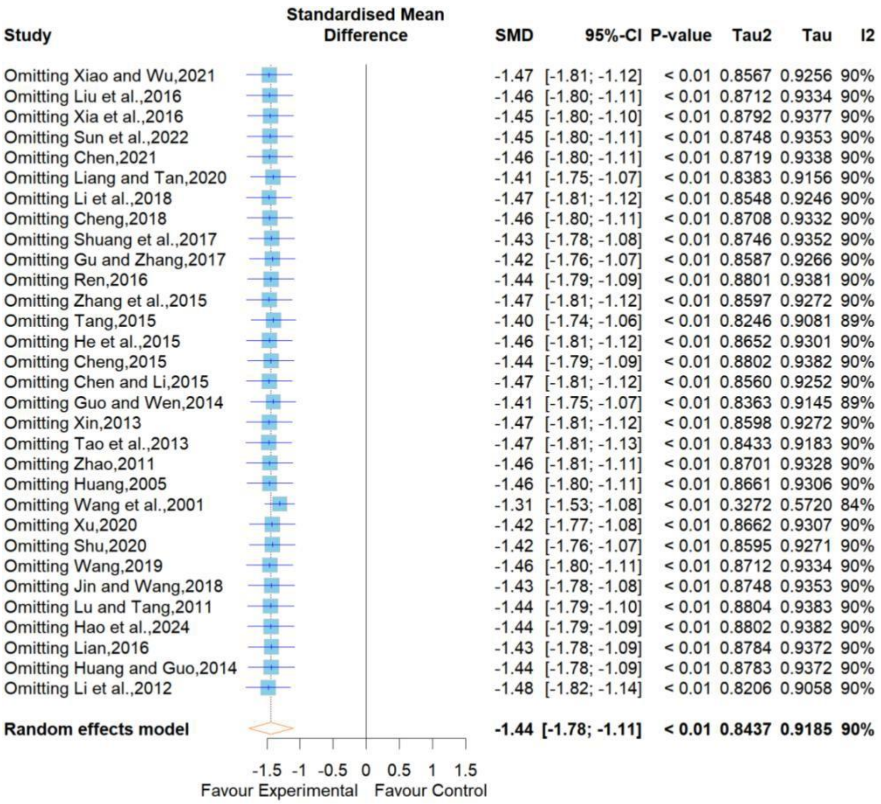

Supplementary Figure S5. Forest plot of sensitivity analysis of Xingnaojing on the Glasgow coma scale.

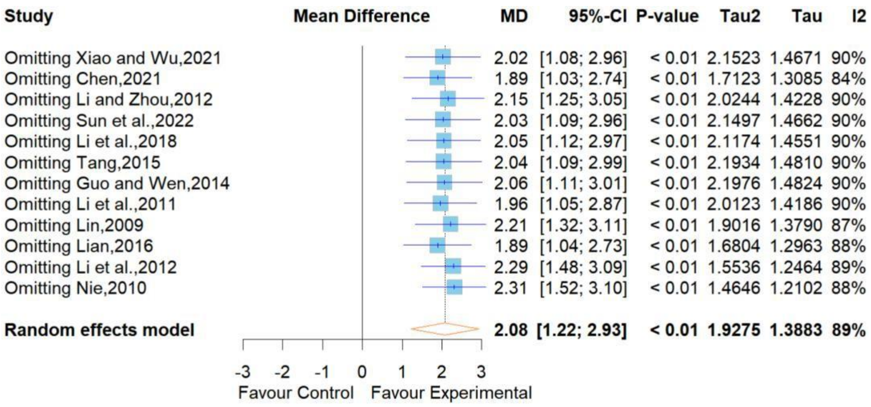

Supplementary Figure S6. Forest plot of sensitivity analysis of Xingnaojing on the activities of daily living.

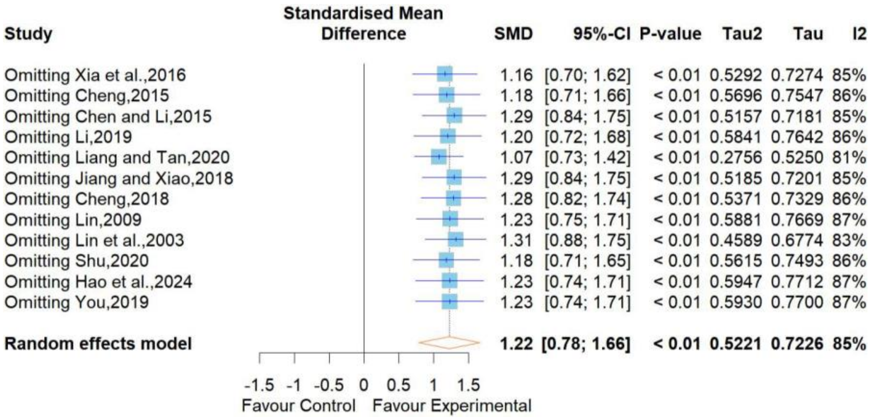

Supplementary Figure S7. Forest plot of sensitivity analysis of Xingnaojing on the intracerebral hematoma volume.

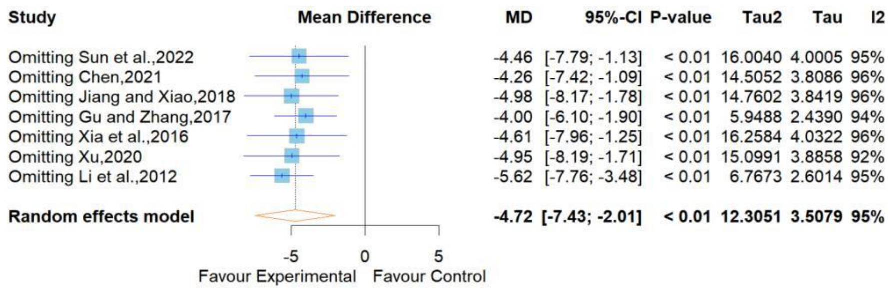

Supplementary Figure S8. Forest plot of sensitivity analysis of Xingnaojing on the volume of perihematomal edema.

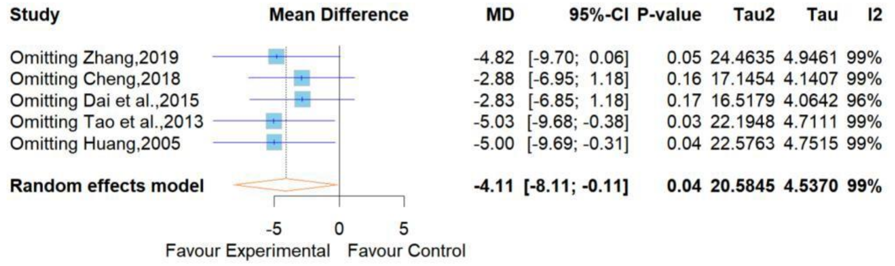

Supplementary Figure S9. Forest plot of sensitivity analysis of Xingnaojing on TNF-a

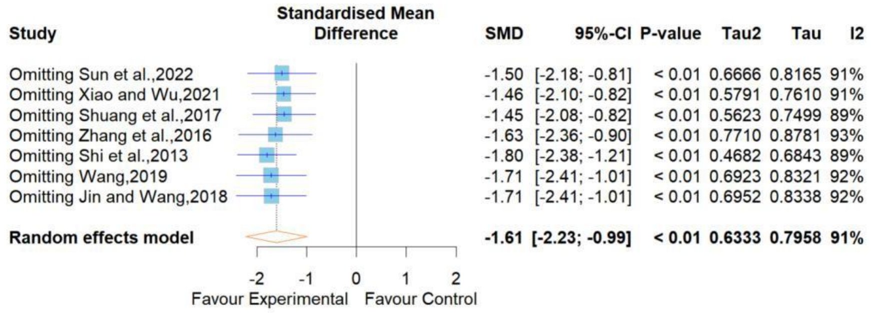

Supplementary Figure S10. Forest plot of sensitivity analysis of Xingnaojing on adverse drug reactions.

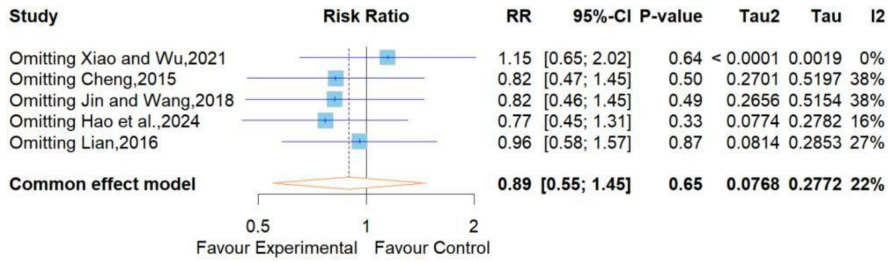

Supplementary Figure S11. Forest plot of sensitivity analysis of Xingnaojing on incidence of complications.

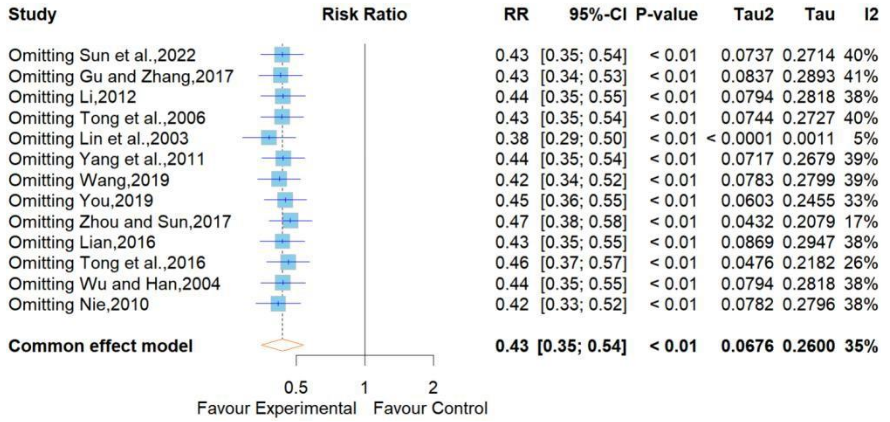

Supplementary Figure 12. Forest plot of Xingnaojing on total efficiency rate assessed by different criteria.

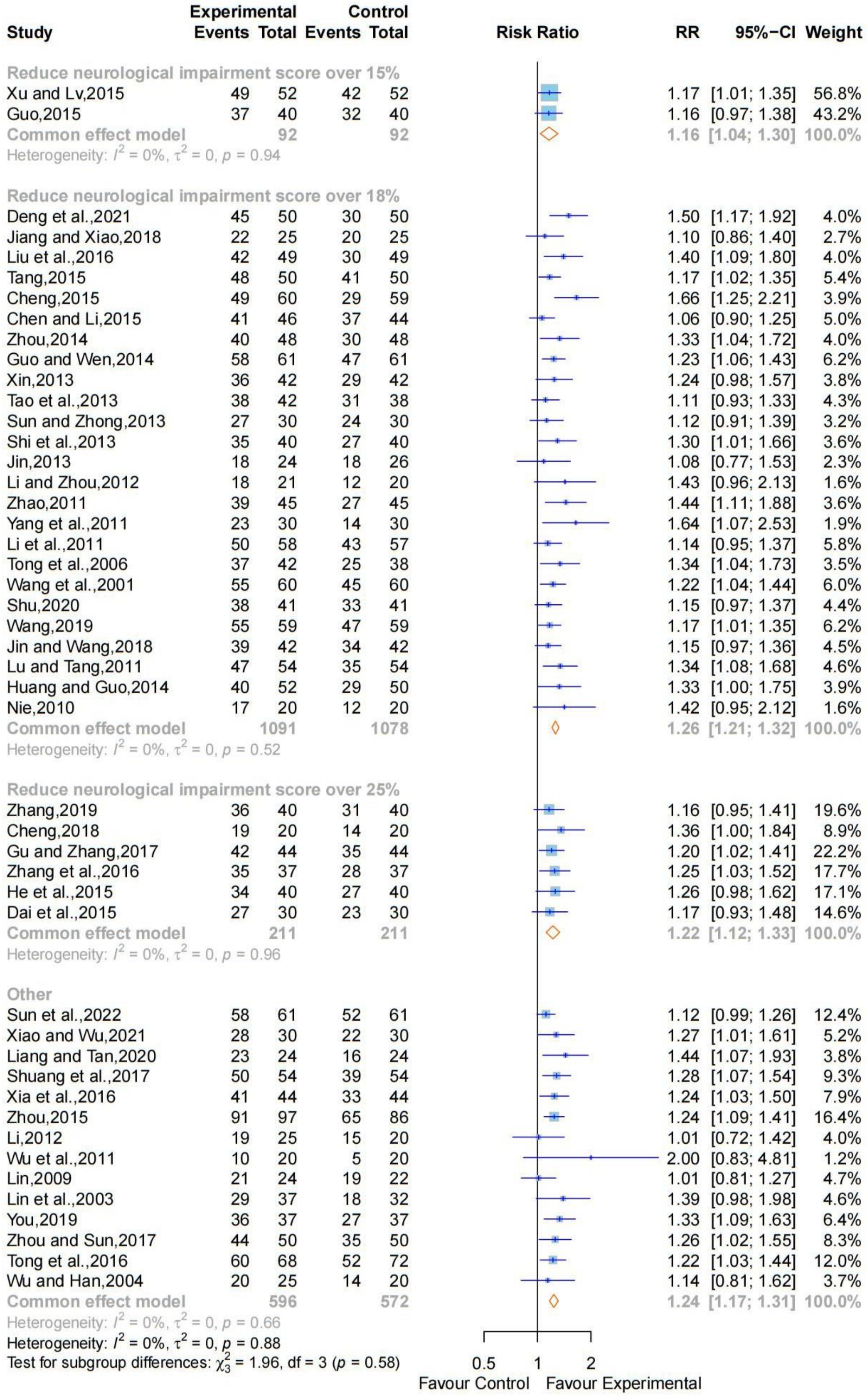

Supplementary Figure S13. Forest plot of Xingnaojing on neurological impairment assessed by different scales (MD).

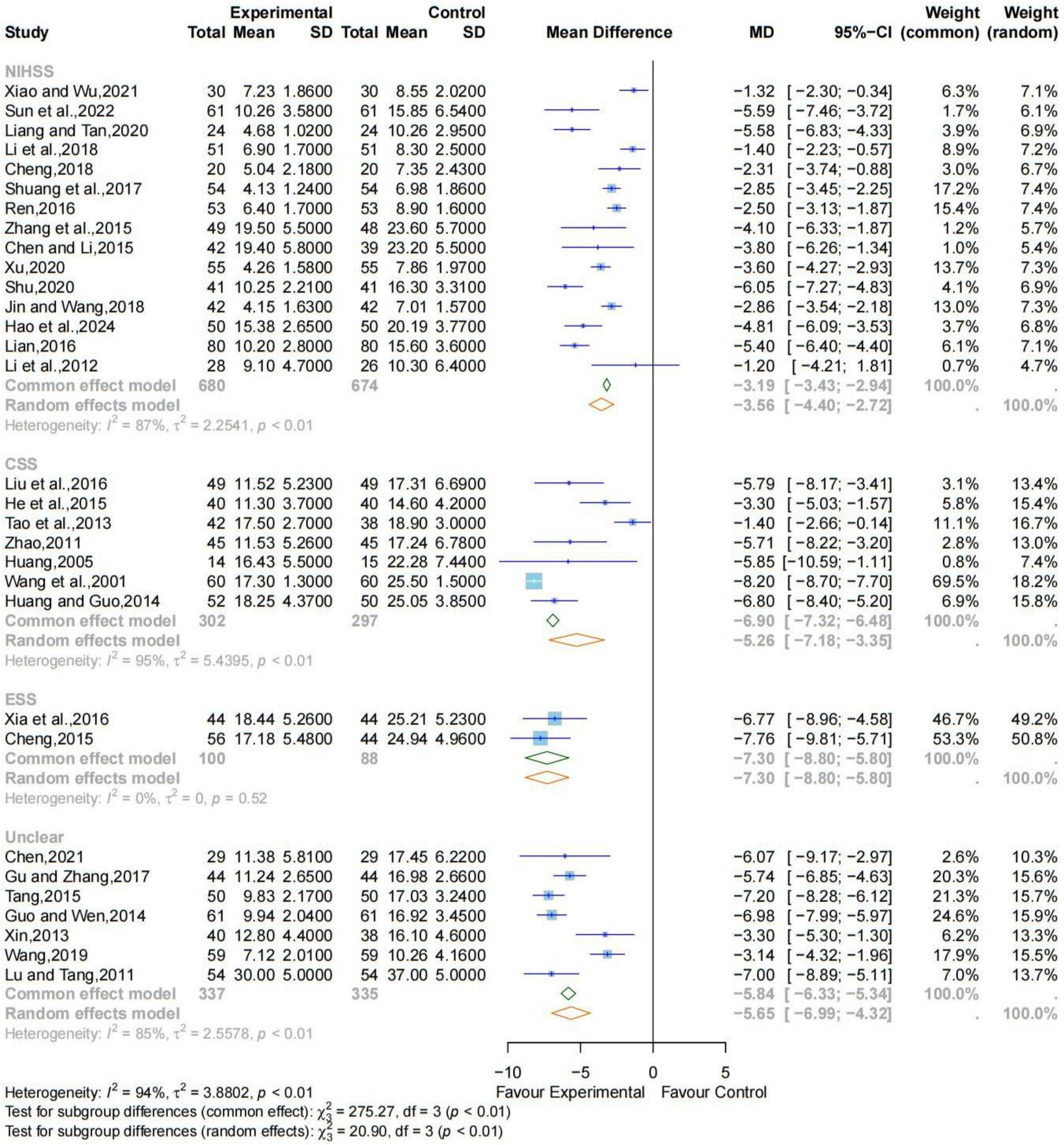

Supplementary Figure S14. Forest plot of Xingnaojing on the Activities of Daily Living assessed by different scales (MD).

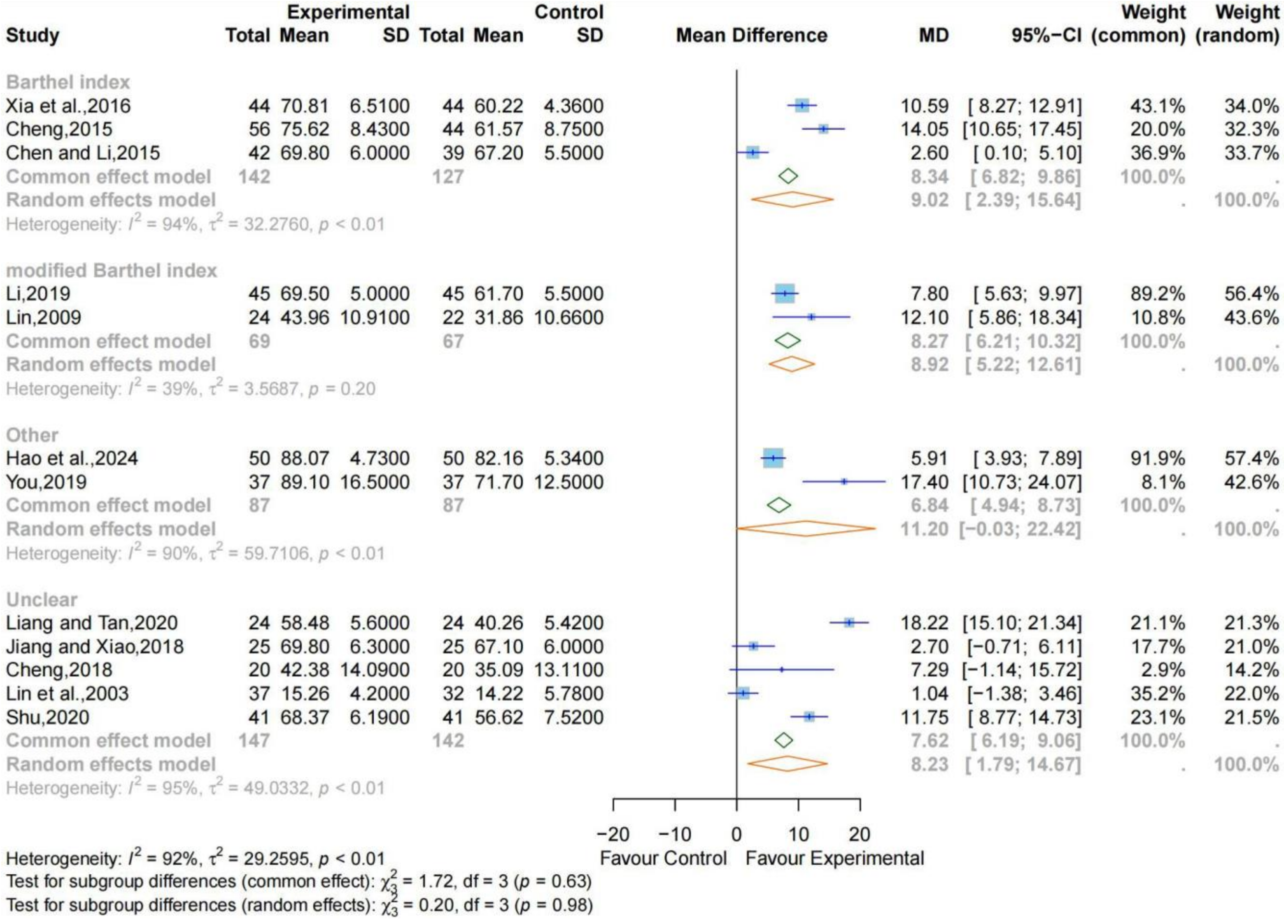

3 Supplementary Files

Supplementary File S1. Search strategy

Searching Strategies (PubMed)

Search number	Query
**1**	"Cerebral Hemorrhage"[Mesh]
**2**	(Hemorrhage, Cerebrum[Title/Abstract]) OR (Cerebrum Hemorrhage[Title/Abstract]) OR (Cerebrum Hemorrhages[Title/Abstract]) OR (Hemorrhages, Cerebrum[Title/Abstract]) OR (Cerebral Parenchymal Hemorrhage[Title/Abstract]) OR (Cerebral Parenchymal Hemorrhages[Title/Abstract]) OR (Hemorrhage, Cerebral Parenchymal[Title/Abstract]) OR (Hemorrhages, Cerebral Parenchymal[Title/Abstract]) OR (Parenchymal Hemorrhage, Cerebral[Title/Abstract]) OR (Parenchymal Hemorrhages, Cerebral[Title/Abstract]) OR (Intracerebral Hemorrhage[Title/Abstract]) OR (Hemorrhage, Intracerebral[Title/Abstract]) OR (Hemorrhages, Intracerebral[Title/Abstract]) OR (Intracerebral Hemorrhages[Title/Abstract]) OR (Hemorrhage, Cerebral[Title/Abstract]) OR (Cerebral Hemorrhages[Title/Abstract]) OR (Hemorrhages, Cerebral[Title/Abstract]) OR (Brain Hemorrhage, Cerebral[Title/Abstract]) OR (Brain Hemorrhages, Cerebral [Title/Abstract]) OR (Cerebral Brain Hemorrhage[Title/Abstract]) OR (Cerebral Brain Hemorrhages[Title/Abstract]) OR (Hemorrhage, Cerebral Brain[Title/Abstract]) OR (Hemorrhages, Cerebral Brain[Title/Abstract])
**3**	#1 OR #2
**4**	"Hemorrhagic Stroke"[Mesh]
**5**	(Hemorrhagic Strokes[Title/Abstract]) OR (Stroke, Hemorrhagic[Title/Abstract]) OR (Subarachnoid Hemorrhagic Stroke[Title/Abstract]) OR (Hemorrhagic Stroke, Subarachnoid[Title/Abstract]) OR (Stroke, Subarachnoid Hemorrhagic[Title/Abstract]) OR (Subarachnoid Hemorrhagic Strokes[Title/Abstract]) OR (Intracerebral Hemorrhagic Stroke[Title/Abstract]) OR (Hemorrhagic Stroke, Intracerebral[Title/Abstract]) OR (Intracerebral Hemorrhagic Strokes[Title/Abstract]) OR (Stroke, Intracerebral Hemorrhagic[Title/Abstract]) OR (Intracerebral Hemorrhage Stroke[Title/Abstract]) OR (Hemorrhage Stroke, Intracerebral[Title/Abstract]) OR (Intracerebral Hemorrhage Strokes[Title/Abstract]) OR (Stroke, Intracerebral Hemorrhage[Title/Abstract])
**6**	#4 OR #5
**7**	#3 OR #6
**8**	"xingnaojing" [Supplementary Concept]
**9**	xingnaojing[Title/Abstract]) OR (xingnaojing inject*[Title/Abstract]) OR (xing-nao-jing[Title/Abstract]) OR (xing-nao-jing inject*[Title/Abstract]) OR (xingnaojing zhushe[Title/Abstract]) OR (xing nao jing zhu she[Title/Abstract]) OR (xing-nao-jing-zhu-she[Title/Abstract]) OR (XNJ[Title/Abstract]) OR (XNJI[Title/Abstract])
**10**	#8 OR #9
**11**	#7 AND #10

Searching Strategies (Embase)

Search number	Query
**1**	**'brain hemorrhage'/exp**
**2**	'bleeding, corpus callosum':ti,ab,kw OR 'brain bleeding':ti,ab,kw OR 'brain haemorrhage':ti,ab,kw OR 'brain haemorrhage, traumatic':ti,ab,kw OR 'brain hemorrhage, traumatic':ti,ab,kw OR 'brain microhaemorrhage':ti,ab,kw OR 'brain microhemorrhage':ti,ab,kw OR 'brain stem haemorrhage, traumatic':ti,ab,kw OR 'brain stem hemorrhage, traumatic':ti,ab,kw OR 'cerebral haemorrhage':ti,ab,kw OR 'cerebral haemorrhage, traumatic':ti,ab,kw OR 'cerebral hemorrhage':ti,ab,kw OR 'cerebral hemorrhage, traumatic':ti,ab,kw OR 'cerebral microbleed':ti,ab,kw OR 'corpus callosum bleeding':ti,ab,kw OR 'corpus callosum haemorrhage':ti,ab,kw OR 'corpus callosum hemorrhage':ti,ab,kw OR 'encephalorrhagia':ti,ab,kw OR 'haemorrhage, brain':ti,ab,kw OR 'haemorrhage, intracranial':ti,ab,kw OR 'haemorrhagic apoplexy':ti,ab,kw OR 'haemorrhagic stroke':ti,ab,kw OR 'haemorrhagic stroke intracerebral bleeding':ti,ab,kw OR 'hematencephalon':ti,ab,kw OR 'hemorrhage, brain':ti,ab,kw OR 'hemorrhage, intracranial':ti,ab,kw OR 'hemorrhagic apoplexy':ti,ab,kw OR 'hemorrhagic stroke':ti,ab,kw OR 'hemorrhagic stroke intracerebral bleeding':ti,ab,kw OR 'hypertensive intracranial haemorrhage':ti,ab,kw OR 'hypertensive intracranial hemorrhage':ti,ab,kw OR 'intracerebral bleeding':ti,ab,kw OR 'intracerebral haemorrhage':ti,ab,kw OR 'intracerebral hemorrhage':ti,ab,kw OR 'intracortical haemorrhage':ti,ab,kw OR 'intracortical hemorrhage':ti,ab,kw OR 'intracranial bleeding':ti,ab,kw OR 'intracranial haemorrhage':ti,ab,kw OR 'intracranial haemorrhage, hypertensive':ti,ab,kw OR 'intracranial haemorrhage, traumatic':ti,ab,kw OR 'intracranial haemorrhages':ti,ab,kw OR 'intracranial hemorrhage':ti,ab,kw OR 'intracranial hemorrhage, hypertensive':ti,ab,kw OR 'intracranial hemorrhage, traumatic':ti,ab,kw OR 'intracranial hemorrhages':ti,ab,kw OR 'intraventricular haemorrhage':ti,ab,kw OR 'intraventricular hemorrhage':ti,ab,kw OR 'periventricular haemorrhage':ti,ab,kw OR 'periventricular hemorrhage':ti,ab,kw OR 'posterior fossa haemorrhage':ti,ab,kw OR 'posterior fossa hemorrhage':ti,ab,kw OR 'traumatic brain haemorrhage':ti,ab,kw OR 'traumatic brain hemorrhage':ti,ab,kw OR 'traumatic brain stem haemorrhage':ti,ab,kw OR 'traumatic brain stem hemorrhage':ti,ab,kw OR 'traumatic cerebral haemorrhage':ti,ab,kw OR 'traumatic cerebral hemorrhage':ti,ab,kw OR 'traumatic intracranial haemorrhage':ti,ab,kw OR 'traumatic intracranial hemorrhage':ti,ab,kw OR 'brain hemorrhage':ti,ab,kw
**3**	#1 OR #2
**4**	'xingnaojing'/exp
**5**	'xing nao jing*':ti,ab,kw OR 'xing nao jing injection*':ti,ab,kw OR 'xing nao jing zhu she*':ti,ab,kw OR 'xing-nao-jing*':ti,ab,kw OR 'xing-nao-jing injection*':ti,ab,kw OR 'xing-nao-jing-zhu-she*':ti,ab,kw OR 'xingnaojing injection*':ti,ab,kw OR 'xingnaojing zhushe*':ti,ab,kw OR 'xingnaojingzhushe*':ti,ab,kw OR 'xingnaojing*':ti,ab,kw OR 'xingnaojing injections*':ti,ab,kw OR 'xing-nao-jing injections*':ti,ab,kw OR 'XNJ*':ti,ab,kw OR 'XNJI':ti,ab,kw
**6**	#3 OR #4
**7**	#3 AND #6

Searching Strategies (the Cochrane library)

Search number	Query
**1**	MeSH descriptor: [Cerebral Hemorrhage] explode all trees
**2**	(Cerebral Parenchymal Hemorrhage):ti,ab,kw OR (Hemorrhages, Cerebral Parenchymal):ti,ab,kw OR (Cerebral Brain Hemorrhage):ti,ab,kw OR (Hemorrhage, Cerebral Brain):ti,ab,kw OR (Hemorrhage, Cerebral Parenchymal):ti,ab,kw OR (Parenchymal Hemorrhages, Cerebral):ti,ab,kw OR (Cerebral Brain Hemorrhages):ti,ab,kw OR (Brain Hemorrhages, Cerebral):ti,ab,kw OR (Hemorrhages, Cerebral Brain):ti,ab,kw OR (Brain Hemorrhage, Cerebral):ti,ab,kw OR (Parenchymal Hemorrhage, Cerebral):ti,ab,kw OR (Cerebral Parenchymal Hemorrhages):ti,ab,kw OR (Hemorrhage, Intracerebral):ti,ab,kw OR (Intracerebral Hemorrhages):ti,ab,kw OR (Hemorrhages, Intracerebral):ti,ab,kw OR (Hemorrhage, Cerebral):ti,ab,kw OR (Hemorrhages, Cerebrum):ti,ab,kw OR (Cerebrum Hemorrhage):ti,ab,kw OR (Intracerebral Hemorrhage):ti,ab,kw OR (Hemorrhages, Cerebral):ti,ab,kw OR (Cerebrum Hemorrhages):ti,ab,kw OR (Cerebral Hemorrhages):ti,ab,kw OR (Hemorrhage, Cerebrum):ti,ab,kw
**3**	#1 OR #2
**4**	MeSH descriptor: [Hemorrhagic Stroke] explode all trees
**5**	(Subarachnoid Hemorrhagic Stroke):ti,ab,kw OR (Hemorrhagic Stroke, Subarachnoid):ti,ab,kw OR (Stroke, Subarachnoid Hemorrhagic):ti,ab,kw OR (Subarachnoid Hemorrhagic Strokes):ti,ab,kw OR (Stroke, Intracerebral Hemorrhagic):ti,ab,kw OR (Intracerebral Hemorrhage Stroke):ti,ab,kw OR (Stroke, Intracerebral Hemorrhage):ti,ab,kw OR (Intracerebral Hemorrhagic Strokes):ti,ab,kw OR (Hemorrhagic Stroke, Intracerebral):ti,ab,kw OR (Intracerebral Hemorrhagic Stroke):ti,ab,kw OR (Hemorrhage Stroke, Intracerebral):ti,ab,kw OR (Intracerebral Hemorrhage Strokes):ti,ab,kw OR (Hemorrhagic Strokes):ti,ab,kw OR (Stroke, Hemorrhagic):ti,ab,kw
**6**	#3 OR #4
**7**	#3 OR #6
**8**	(xingnaojing):ti,ab,kw OR (xingnaojing inject*):ti,ab,kw OR (xing-nao-jing):ti,ab,kw OR (xing-nao-jing inject*):ti,ab,kw OR (xingnaojing zhushe):ti,ab,kw OR (xing nao jing zhu she):ti,ab,kw OR (xing-nao-jing-zhu-she):ti,ab,kw OR (XNJ):ti,ab,kw OR (XNJI):ti,ab,kw
**9**	#7 AND #8

Searching Strategies (Web of Science)

(TS=(Cerebral Hemorrhage) OR TS=(Hemorrhage, Cerebrum) OR TS=(Cerebrum Hemorrhage) OR TS=(Cerebrum Hemorrhages) OR TS=(Hemorrhages, Cerebrum) OR TS=(Cerebral Parenchymal Hemorrhage) OR TS=(Cerebral Parenchymal Hemorrhages) OR TS=(Hemorrhage, Cerebral Parenchymal) OR TS=(Hemorrhages, Cerebral Parenchymal) OR TS=(Parenchymal Hemorrhage, Cerebral) OR TS=(Parenchymal Hemorrhages, Cerebral) OR TS=(Intracerebral Hemorrhage) OR TS=(Hemorrhage, Intracerebral) OR TS=(Hemorrhages, Intracerebral) OR TS=(Intracerebral Hemorrhages) OR TS=(Hemorrhage, Cerebral) OR TS=(Cerebral Hemorrhages) OR TS=(Hemorrhages, Cerebral) OR TS=(Brain Hemorrhage, Cerebral) OR TS=(Brain Hemorrhages, Cerebral) OR TS=(Cerebral Brain Hemorrhage) OR TS=(Cerebral Brain Hemorrhages) OR TS=(Hemorrhage, Cerebral Brain) OR TS=(Hemorrhages, Cerebral Brain) OR TS=(Hemorrhagic Stroke) OR TS=(Hemorrhagic Strokes) OR TS=(Stroke, Hemorrhagic) OR TS=(Subarachnoid Hemorrhagic Stroke) OR TS=(Hemorrhagic Stroke, Subarachnoid) OR TS=(Stroke, Subarachnoid Hemorrhagic) OR TS=(Subarachnoid Hemorrhagic Strokes) OR TS=(Intracerebral Hemorrhagic Stroke) OR TS=(Hemorrhagic Stroke, Intracerebral) OR TS=(Intracerebral Hemorrhagic Strokes) OR TS=(Stroke, Intracerebral Hemorrhagic) OR TS=(Intracerebral Hemorrhage Stroke) OR TS=(Hemorrhage Stroke, Intracerebral) OR TS=(Intracerebral Hemorrhage Strokes) OR TS=(Stroke, Intracerebral Hemorrhage)) AND (TS=(xingnaojing) OR TS=(xingnaojing inject*) OR TS=(xing-nao-jing) OR TS=(xing-nao-jing inject*) OR TS=(xingnaojing zhushe) OR TS=(xing nao jing zhu she) OR TS=(xing-nao-jing-zhu-she) OR TS=(XNJ) OR TS=(XNJI))

Searching Strategies (CNKI)

(SU %= '脑出血' OR SU %= '脑溢血' OR SU %= '脑实质内出血' OR SU %= '脑实质出血' OR SU %= '颅内出血' OR SU %= '颅内血肿' OR SU %= '脑内血肿' OR SU %= '出血性中风' OR SU %= '出血性脑中风' OR SU %= '出血性卒中' OR SU %= '出血性脑卒中' OR SU %= '小脑出血' OR SU %= '脑叶出血' OR SU %= '基底节出血' OR SU %= '基底节区出血' OR SU %= '脑干出血' OR SU %= '幕上出血' OR SU %= '脑室出血') AND (TKA = '醒脑静' OR TKA = '醒脑静注射液' OR TKA = '醒脑静注射剂' OR TKA = '醒脑静注射' OR TKA = '醒脑净' OR TKA = '醒脑净注射液' OR TKA = '醒脑净注射剂' OR TKA = '醒脑净注射' OR TKA = 'XNJ' OR TKA = 'XNJI' OR TKA = 'xingnaojing' OR TKA = 'xing-nao-jing')

Searching Strategies (WanFang)

主题:("脑出血" or "脑溢血" or "脑实质内出血" or "脑实质出血" or "颅内出血" or "颅内血肿" or "出血性中风" or "出血性脑中风" or "出血性卒中" or "出血性脑卒中" or "小脑出血" or "脑叶出血" or "基底节出血" or "基底节区出血" or "脑干出血" or "脑内血肿" or "幕上出血" or "脑室出血") and 主题:("醒脑静" or "醒脑静注射液" or "醒脑静注射剂" or "醒脑静注射" or "醒脑净" or "醒脑净注射液" or "醒脑净注射剂" or "醒脑净注射" or "XNJ" or "XNJI" or "xingnaojing" or "xing-nao-jing")

Searching Strategies (VIP Database)

M=(脑出血 + 脑溢血 + 脑实质内出血 + 脑实质出血 + 颅内出血 + 颅内血肿 + 出血性脑卒中 + 出血性卒中 + 出血性脑中风 + 出血性中风 + 小脑出血 + 脑叶出血 + 基底节出血 + 基底节区出血 + 脑干出血 + 脑内血肿 + 幕上出血 + 脑室出血) AND U=(醒脑静 + 醒脑静注射液 + 醒脑静注射剂 + 醒脑静注射 + 醒脑净 + 醒脑净注射液 + 醒脑净注射剂 + 醒脑净注射 + XNJ + XNJI + xingnaojing + xing-nao-jing)

Searching Strategies (SinoMed)

("脑出血"[不加权:扩展] OR "出血性卒中"[不加权:扩展] OR "脑溢血"[核心字段:智能] OR "脑实质内出血"[核心字段:智能] OR "脑实质出血"[核心字段:智能] OR "颅内出血"[核心字段:智能] OR "颅内血肿"[核心字段:智能] OR "出血性中风"[核心字段:智能] OR "出血性脑中风"[核心字段:智能] OR "出血性脑卒中"[核心字段:智能] OR "小脑出血"[核心字段:智能] OR "脑叶出血"[核心字段:智能] OR "基底节出血"[核心字段:智能] OR "基底节区出血"[核心字段:智能] OR "脑干出血"[核心字段:智能] OR "脑内血肿"[核心字段:智能] OR "幕上出血"[核心字段:智能] OR "脑室出血"[核心字段:智能]) AND ("醒脑静注射液"[不加权:扩展] OR "醒脑静"[常用字段:智能] OR "醒脑静注射剂"[常用字段:智能] OR "醒脑静注射"[常用字段:智能] OR "醒脑净"[常用字段:智能] OR "醒脑净注射液"[常用字段:智能] OR "醒脑净注射剂"[常用字段:智能] OR "醒脑净注射"[常用字段:智能] OR "XNJ"[常用字段:智能] OR "XNJI"[常用字段:智能] OR "xingnaojing"[常用字段:智能] OR "xing-nao-jing"[常用字段:智能])

Supplementary File S2. The PRISMA checklist of this meta-analysis

Section and Topic	Item #	Checklist item	Location where item is reported
**TITLE**			
Title	1	Identify the report as a systematic review.	
**ABSTRACT**			
Abstract	2	See the PRISMA 2020 for Abstracts checklist.	
**INTRODUCTION**			
Rationale	3	Describe the rationale for the review in the context of existing knowledge.	
Objectives	4	Provide an explicit statement of the objective(s) or question(s) the review addresses.	
**METHODS**			
Eligibility criteria	5	Specify the inclusion and exclusion criteria for the review and how studies were grouped for the syntheses.	
Information sources	6	Specify all databases, registers, websites, organisations, reference lists and other sources searched or consulted to identify studies. Specify the date when each source was last searched or consulted.	
Search strategy	7	Present the full search strategies for all databases, registers and websites, including any filters and limits used.	Supplementary File S1
Selection process	8	Specify the methods used to decide whether a study met the inclusion criteria of the review, including how many reviewers screened each record and each report retrieved, whether they worked independently, and if applicable, details of automation tools used in the process.	Figure 1
Data collection process	9	Specify the methods used to collect data from reports, including how many reviewers collected data from each report, whether they worked independently, any processes for obtaining or confirming data from study investigators, and if applicable, details of automation tools used in the process.	
Data items	10a	List and define all outcomes for which data were sought. Specify whether all results that were compatible with each outcome domain in each study were sought (e.g. for all measures, time points, analyses), and if not, the methods used to decide which results to collect.	Table1
	10b	List and define all other variables for which data were sought (e.g. participant and intervention characteristics, funding sources). Describe any assumptions made about any missing or unclear information.	
Study risk of bias assessment	11	Specify the methods used to assess risk of bias in the included studies, including details of the tool(s) used, how many reviewers assessed each study and whether they worked independently, and if applicable, details of automation tools used in the process.	
Effect measures	12	Specify for each outcome the effect measure(s) (e.g. risk ratio, mean difference) used in the synthesis or presentation of results.	
Synthesis	13a	Describe the processes used to decide which studies were eligible for each synthesis (e.g. tabulating the study intervention	

Section and Topic	Item #	Checklist item	Location where item is reported
methods		characteristics and comparing against the planned groups for each synthesis (item #5)).	
	13b	Describe any methods required to prepare the data for presentation or synthesis, such as handling of missing summary statistics, or data conversions.	
	13c	Describe any methods used to tabulate or visually display results of individual studies and syntheses.	
	13d	Describe any methods used to synthesize results and provide a rationale for the choice(s). If meta-analysis was performed, describe the model(s), method(s) to identify the presence and extent of statistical heterogeneity, and software package(s) used.	
	13e	Describe any methods used to explore possible causes of heterogeneity among study results (e.g. subgroup analysis, meta-regression).	
	13f	Describe any sensitivity analyses conducted to assess robustness of the synthesized results.	
Reporting bias assessment	14	Describe any methods used to assess risk of bias due to missing results in a synthesis (arising from reporting biases).	
Certainty assessment	15	Describe any methods used to assess certainty (or confidence) in the body of evidence for an outcome.	
**RESULTS**			
Study selection	16a	Describe the results of the search and selection process, from the number of records identified in the search to the number of studies included in the review, ideally using a flow diagram.	Figure 1
	16b	Cite studies that might appear to meet the inclusion criteria, but which were excluded, and explain why they were excluded.	N/A
Study characteristics	17	Cite each included study and present its characteristics.	Table1
Risk of bias in studies	18	Present assessments of risk of bias for each included study.	Figure 2 and Supplementary Figure S1
Results of individual studies	19	For all outcomes, present, for each study: (a) summary statistics for each group (where appropriate) and (b) an effect estimate and its precision (e.g. confidence/credible interval), ideally using structured tables or plots.	Figures 3-10
Results of syntheses	20a	For each synthesis, briefly summarise the characteristics and risk of bias among contributing studies.	Figures 3-10
	20b	Present results of all statistical syntheses conducted. If meta-analysis was done, present for each the summary estimate and its precision (e.g. confidence/credible interval) and measures of statistical heterogeneity. If comparing groups, describe the direction of the effect.	Figures 3-10
	20c	Present results of all investigations of possible causes of heterogeneity among study results.	Figures 3-10, Supplementary

Section and Topic	Item #	Checklist item	Location where item is reported
			Tables S1-S6
	20d	Present results of all sensitivity analyses conducted to assess the robustness of the synthesized results.	Supplementary Figures S2-S8
Reporting biases	21	Present assessments of risk of bias due to missing results (arising from reporting biases) for each synthesis assessed.	N/A
Certainty of evidence	22	Present assessments of certainty (or confidence) in the body of evidence for each outcome assessed.	Table 3
**DISCUSSION**			
Discussion	23a	Provide a general interpretation of the results in the context of other evidence.	
	23b	Discuss any limitations of the evidence included in the review.	
	23c	Discuss any limitations of the review processes used.	
	23d	Discuss implications of the results for practice, policy, and future research.	
**OTHER INFORMATION**			
Registration and protocol	24a	Provide registration information for the review, including register name and registration number, or state that the review was not registered.	
	24b	Indicate where the review protocol can be accessed, or state that a protocol was not prepared.	
	24c	Describe and explain any amendments to information provided at registration or in the protocol.	N/A
Support	25	Describe sources of financial or non-financial support for the review, and the role of the funders or sponsors in the review.	
Competing interests	26	Declare any competing interests of review authors.	
Availability of data, code and other materials	27	Report which of the following are publicly available and where they can be found: template data collection forms; data extracted from included studies; data used for all analyses; analytic code; any other materials used in the review.	

From: Page MJ, McKenzie JE, Bossuyt PM, Boutron I, Hoffmann TC, Mulrow CD, et al. The PRISMA 2020 statement: an updated guideline for reporting systematic reviews. BMJ 2021;372:n71. doi: 10.1136/bmj.n71

For more information, visit: http://www.prisma-statement.org/
